# Experimental vitamin B_12_ deficiency in a human subject: a longitudinal investigation of the performance of the holotranscobalamin (HoloTC, Active-B12) immunoassay

**DOI:** 10.1186/s40064-016-1740-5

**Published:** 2016-02-25

**Authors:** Paul Henry Golding

**Affiliations:** Unit 5, 18 Webster Road, Nambour, QLD 4560 Australia

**Keywords:** Holotranscobalamin, HoloTC, Active-B12, Vitamin B_12_, Methylmalonic acid, Self-experimentation

## Abstract

Based on Victor Herbert’s model for sequential stages in the development of vitamin B_12_ deficiency, the holotranscobalamin (HoloTC) immunoassay has controversially been promoted as a more specific and sensitive replacement for the total vitamin B_12_ test, for the diagnosis of deficiency. There have been no longitudinal studies, by means of experimental cobalamin deficiency, because ethical considerations prevent such risky studies on patients or healthy human volunteers. The objective was to provide a detailed record of the response of HoloTC, compared to total vitamin B_12_ and metabolites, to the development of experimental vitamin B_12_ deficiency in an initially replete human subject. This 54 year old male, with a vitamin B_12_ deficiency possibly caused by a defect in the intracellular cobalamin metabolism, ensured an initially replete condition by means of oral doses of cyanocobalamin supplements at 1000 μg/day for 12 weeks. The subject then depleted himself of vitamin B_12_, by withholding treatment and using a low-cobalamin diet, until significant metabolic disturbances were observed. The responses of serum total vitamin B_12_ and HoloTC and the two metabolites, plasma methylmalonic acid and homocysteine, were monitored by weekly blood tests. HoloTC was not significantly more sensitive than either total serum vitamin B_12_ or total homocysteine, and was much less sensitive than methylmalonic acid. HoloTC decreased from an initial concentration of >128 pmol/L to a minimum of 33 pmol/L on day 742, the only day on which it fell below the lower limit of the reference interval. Total vitamin B_12_ decreased from an initial concentration of 606 pmol/L to a minimum of 171 pmol/L on day 728. Total homocysteine increased from an initial concentration of 8.4 μmol/L to a maximum of 14.2 μmol/L on day 609. Methylmalonic acid unexpectedly contained four distinct peaks; initially at 0.17 μmol/L, it first exceeded the upper limit of the reference interval on day 386, finally reaching a maximum peak of 0.90 μmol/L on day 658. The results of this experiment are inconsistent with Herbert’s hypothesis that HoloTC is the earliest marker of vitamin B_12_ deficiency, and therefore do not support his model for the staged development of vitamin B_12_ deficiency.

**Electronic supplementary material:**

The online version of this article (doi:10.1186/s40064-016-1740-5) contains supplementary material, which is available to authorized users.

## Background

Based on Victor Herbert’s model for sequential stages in the development of vitamin B_12_ deficiency (Herbert 1987a, 1994), the holotranscobalamin (HoloTC) immunoassay has controversially been promoted as a more specific and sensitive replacement for the total vitamin B_12_ test, for the diagnosis of deficiency (Axis-Shield and Abbott Laboratories 2006a, b, 2007a, b; Axis-Shield 2007a, b, 2012, 2014a, b).

Other researchers have reviewed the HoloTC immunoassay (Morkbak et al. 2005; Aparicio-Ugarriza et al. 2014). This author’s detailed review of Herbert’s model and the HoloTC immunoassay, and suggested alternative hypothesis for the development of vitamin B_12_ deficiency, has been submitted to this journal as a separate article. Although there have been many published reports supporting the use of the HoloTC immunoassay, several experimenters reported results that do not support the claim that HoloTC is the earliest and most sensitive indicator of vitamin B_12_ deficiency (Miller et al. 2005; Clarke et al. 2007; Schrempf et al. 2011; Palacios et al. 2013; Remacha et al. 2014).

There have been no longitudinal studies, by means of experimental cobalamin deficiency, because ethical considerations prevent such risky studies on patients or healthy human volunteers.

Commencing in March 2007, this author used himself as the subject of an experiment to investigate the sensitivity of total vitamin B_12_, HoloTC and the two metabolites, methylmalonic acid (MMA) and total homocysteine (tHcy), to the onset of vitamin B_12_ deficiency.

## Objective

The objective of this study was to investigate the performance of the HoloTC (Active-B12) immunoassay during the development of experimental vitamin B_12_ deficiency in an initially replete human subject, by means of self-experimentation.

## Methods

### Ethics statement

As a member of Committee on Publication Ethics (COPE), this journal requires that experiments on human subjects adhere to the ethical standards of the Declaration of Helsinki. In particular, there must be informed consent of subjects, and the experiment must be approved and overseen by a research ethics committee or institutional review board. Because this author did not obtain informed consent, and the study did not receive ethics committee approval, it is necessary for the author to explain the reasons why publication of this report is ethical.

Firstly, the author was both the experimenter and the single subject, so the requirement for informed consent does not apply. There was no institutional involvement, so there was no possibility of coercion. The subject was assessed by a psychiatrist, a Fellow of the Royal Australian and New Zealand College of Psychiatrists, before the experiment commenced, and found to be competent to evaluate the risks and benefits, and to accept full responsibility for the conduct of the experiment.

Secondly, the Declaration of Helsinki is silent on self-experimentation, because it is concerned with the conduct of research on patients or healthy volunteers by others. The requirement for ethics committee approval therefore does not apply where the single subject is also the sole experimenter. Also, because there was no institution involved in the study, with the experiment conducted by an independent researcher, no ethics committee existed.

Thirdly, the experiment was not performed recklessly or carelessly; the subject’s condition was monitored weekly by a general practitioner and the psychiatrist; neither had any conflict of interests. Being qualified medical practitioners receiving all weekly pathology reports, both doctors were able to continually assess the condition of the subject. The subject instigated, designed and performed the experiment, and the doctors’ only role was monitoring for safety.

Lastly, the motivation for performing the experiment was ethical, and involved no conflict of interests. The author wanted to investigate the performance of the new HoloTC (Active B12) immunoassay, for the diagnosis of vitamin B_12_ deficiency, because he was aware of the potential consequences of misdiagnosis. The author was motivated only by the desire to gain and share knowledge, to advance medical science, for the benefit of patients.

### Experiment design

A human subject, initially replete in vitamin B_12_, consumed a low-cobalamin diet and gradually ceased taking vitamin B_12_ supplements to deplete the body of vitamin B_12_, culminating in significant metabolic disturbances. The responses of serum total vitamin B_12_ and HoloTC and the two metabolites, plasma MMA and tHcy, were monitored by routine blood tests. All tests were performed by pathology laboratories accredited by the Australian National Association of Testing Authorities (NATA). The experiment commenced on day 0 (5 March 2007) and ended on day 854 (6 July 2009).

### The subject

The subject was this author, a 54 year old male non-drinker and non-smoker; he had for many years consumed a lacto-vegetarian diet. The subject was diagnosed with vitamin B_12_ deficiency in October 2005, based on low serum vitamin B_12_ concentration, chronic symptoms of abnormal sensations in the extremities and results of a neurological examination. Because of his history of vitamin B_12_ deficiency, possibly caused by a defect in the intracellular cobalamin metabolism, the subject had been taking 1000 μg oral cyanocobalamin daily for 12 weeks immediately prior to this study. Previous testing showed normal results for serum vitamin B_12_ and the two metabolites, homocysteine and MMA, at this level of supplementation.

### The low-vitamin B_12_ diet

The low-vitamin B_12_ diet was the subject’s usual vegetarian diet. The only source of vitamin B_12_ in the diet was the estimated 1.8 μg/day contained in the 300 ml of milk added to his breakfast cereal (Food Standards Australia and New Zealand 2013). Although this dietary intake would be adequate to maintain the vitamin B_12_ store in a healthy individual (Herbert 1987b), extensive previous testing in his self-experiment of 2005 showed that this subject would become depleted without supplements. In that unpublished experiment, the subject’s serum total vitamin B_12_ concentration decreased, and his MMA and tHcy concentrations increased, after ceasing oral cyanocobalamin treatment.

### Nutritional precautions

Because the subject maintained his normal vegetarian diet, no special nutritional precautions were required. There were no confounding effects of deficiency of folate or other vitamins, minerals or protein. Previous testing showed normal electrolytes, liver function and red-cell haematology.

### Changes in vitamin B_12_ supplementation

**Fig. 1:**
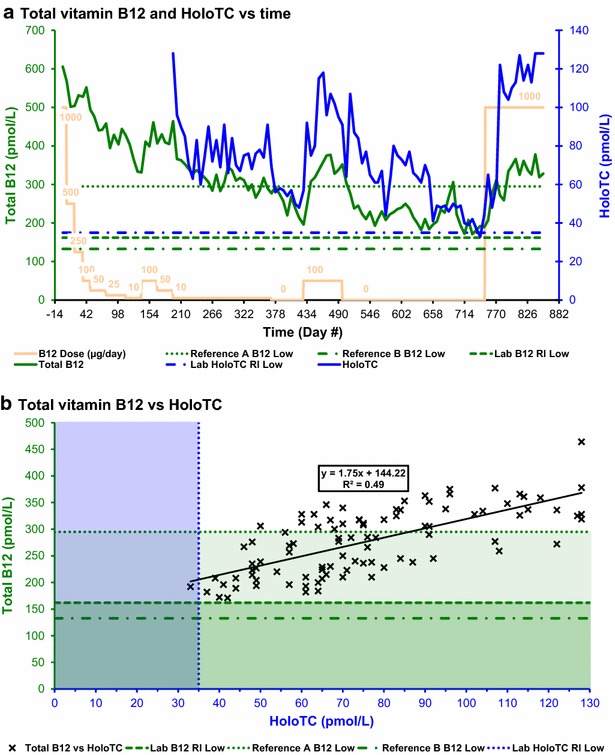
Serum total vitamin B_12_ and HoloTC. **a** Serum total vitamin B_12_ and HoloTC versus time. **b** Serum total vitamin B_12_ versus HoloTC. Reference A B_12_ low = low limit for serum vitamin B_12_ concentration defined by Oh and Brown (2003). Reference B B_12_ low = low limit for serum vitamin B_12_ concentration defined by Bates and Lewis (2012). Lab B_12_ RI low = lower limit of serum vitamin B_12_ concentration reference interval defined by the testing laboratory. Lab HoloTC RI low = lower limit of serum holotranscobalamin concentration reference interval defined by the testing laboratory

At day 0 the subject had been consuming 1000 μg oral cyanocobalamin daily for 12 weeks. On day 7, the supplementation was reduced in steps to reach 10 μg on day 112 (Fig. 1a). The dose was then increased to 100 μg for 4 weeks, to test for the effect on the vitamin B_12_ and metabolite assays, then reduced in steps to reach 0 μg on day 371. The dose was then increased to 100 μg for 10 weeks, to test for the effect on the vitamin B_12_, HoloTC and metabolite assays, then reduced in a single step to 0 μg on day 497. The dose remained at 0 μg until the end of the vitamin B_12_ depletion period on day 751.

### Blood sampling

#### Timing of vitamin B_12_ supplementation and blood sampling

Blood samples were collected 24 h after the last oral vitamin B_12_ supplement was taken by the subject.

#### Blood sample collection, handling and transport

Precautions were taken to ensure that consistent and valid blood samples were received by the laboratories. The commercial clinical laboratory that performed the serum vitamin B_12_, HoloTC and haematology tests collected their own samples, using professional phlebotomists at a government-approved collection centre. The same collection centre also collected and froze plasm samples for assay of the metabolites, homocysteine and MMA, by the NSW Biochemical Genetics Service; a specialised laboratory. The subject always fasted overnight, and was well hydrated, ensuring maximum possible consistency between samples. The phlebotomy technique was chosen to provide the highest quality samples; tourniquet application was carefully controlled, and discard tubes were used where required. Samples were promptly transported from the collection centre to the commercial clinical laboratory, cooled on ice, to avoid deterioration. The frozen plasma samples for metabolite assays were transported, packed in dry ice, by specialised courier.

#### Blood sampling frequency

Blood samples for the vitamin B_12_ and HoloTC were collected weekly. The frequency of blood sampling for the metabolites was adapted according to the rate of change of the MMA and homocysteine responses. Blood samples were initially collected four-weekly; this was increased to fortnightly and then weekly as the rate of change increased.

### Vitamin B_12_ immunoassays

#### Serum total vitamin B_12_ immunoassay

Vitamin B_12_ was assayed weekly by the commercial clinical pathology laboratory, using the Siemens Advia Centaur immunoassay system with the ADVIA Centaur and ACS:180 Vitamin B12 Assay reagent kit (Siemens Healthcare Diagnostics 2007). The laboratory reference interval was given as 162–811 pmol/L and the quoted precision was 10 %.

#### Serum holotranscobalamin (HoloTC) immunoassay

HoloTC was assayed weekly by the commercial clinical pathology laboratory, using the Abbott AxSYM immunoassay analyser with the Axis-Shield AxSYM Active-B12 reagent kit (Axis-Shield and Abbott Laboratories 2007a). The laboratory reference interval was given as >35 pmol/L, with a maximum reportable concentration of 128 pmol/L; the quoted precision was 7 %.

### Vitamin B_12_ metabolite assays

#### Plasma methylmalonic acid (MMA)

Plasma MMA was assayed at intervals from four-weekly to weekly, by the NSW Biochemical Genetics Service, using tandem mass spectrometry. The measurement uncertainty was 12 % and reference interval was 0.06–0.34 μmol/L.

#### Plasma total homocysteine (tHcy)

Plasma tHcy was assayed at intervals from four-weekly to weekly, by the NSW Biochemical Genetics Service, using tandem mass spectrometry. The measurement uncertainty was 9 % and reference interval was 4.8–13.7 μmol/L.

### Haematology

Cell counts were performed by the commercial clinical pathology laboratory using the Sysmex XE-2100 Automated Haematology System.

## Results

### Data availability

The data sets supporting all results are included in a Microsoft Excel spreadsheet file, Additional file 1: Table S1, containing charts and tables. High-resolution images for Figs. 1, 2, 3, 4, 5, 6 and 7 are included in a PDF file, Additional file 2: Figure S1, and a Microsoft PowerPoint file, Additional file 3: Figure S2.

**Fig. 2:**
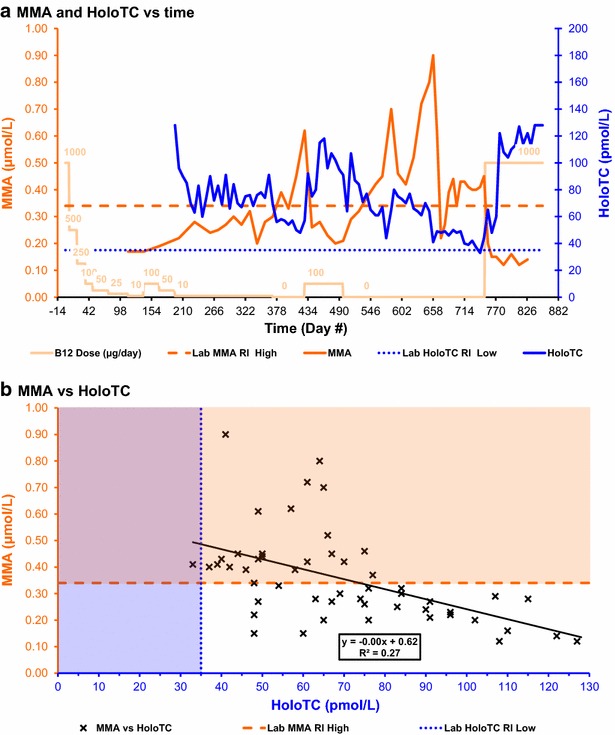
MMA and HoloTC. **a** MMA and HoloTC versus time. **b** MMA versus HoloTC. Lab MMA RI high = upper limit of plasma methylmalonic acid concentration reference interval defined by the testing laboratory. Lab HoloTC RI low = lower limit of serum holotranscobalamin concentration reference interval defined by the testing laboratory

**Fig. 3:**
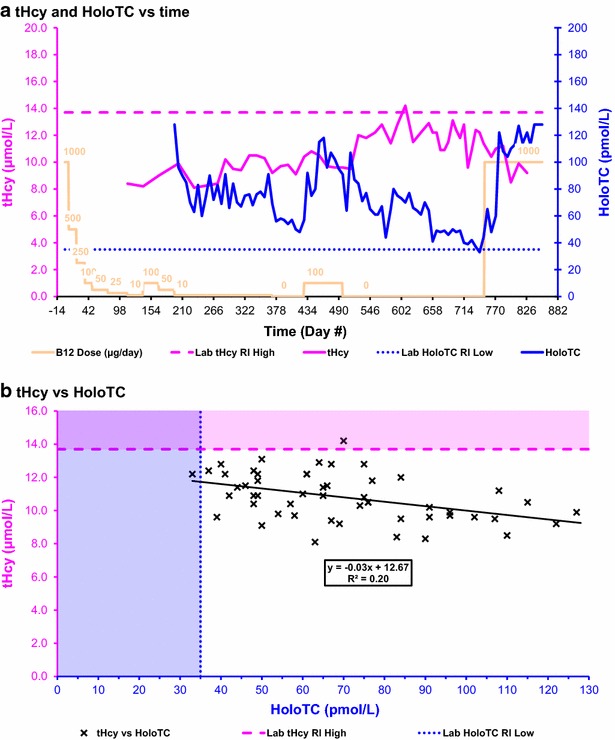
tHcy and HoloTC. **a** tHcy and HoloTC versus time. **b** tHcy versus HoloTC. Lab tHcy RI high = upper limit of plasma total homocysteine concentration reference interval defined by the testing laboratory. Lab HoloTC RI low = lower limit of serum holotranscobalamin concentration reference interval defined by the testing laboratory

**Fig. 4:**
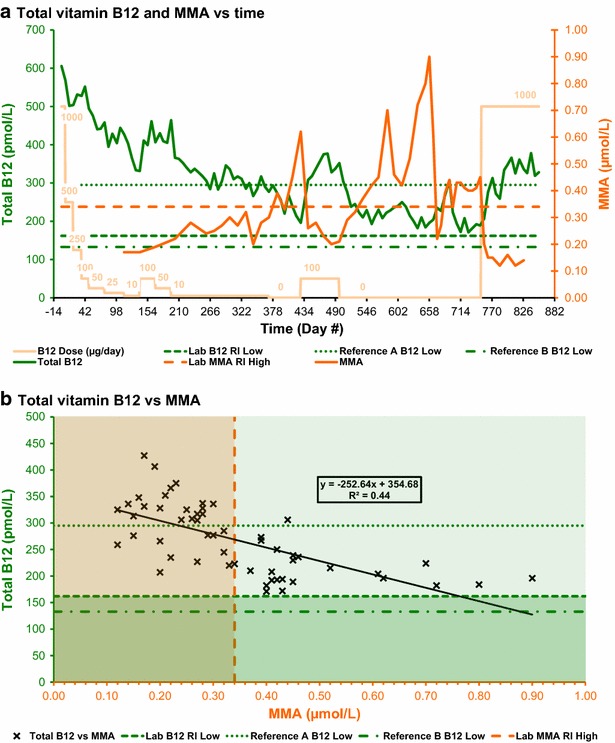
Serum total vitamin B_12_ and MMA. **a** Serum total vitamin B_12_ and MMA versus time. **b** Serum total vitamin B_12_ versus MMA. Reference A B_12_ Low = low limit for serum vitamin B_12_ concentration defined by Oh and Brown (2003). Reference B B_12_ Low = low limit for serum vitamin B_12_ concentration defined by Bates and Lewis (2012). Lab B_12_ RI low = lower limit of serum vitamin B_12_ concentration reference interval defined by the testing laboratory. Lab MMA RI high = upper limit of plasma methylmalonic acid concentration reference interval defined by the testing laboratory

**Fig. 5:**
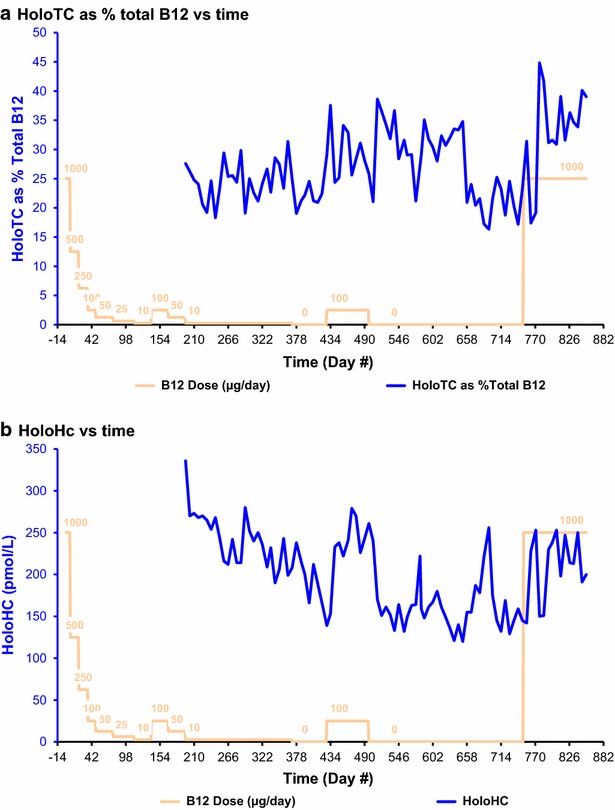
HoloTC and Holohaptocorrin. In **a**, for each pair of concurrent measurements, HoloTC as % of total serum vitamin B_12_ = 100 × (HoloTC concentration/total vitamin B_12_ concentration). In **b**, for each pair of concurrent measurements, holohaptocorrin (HoloHC) = total vitamin B_12_ concentration − holotranscobalamin (HoloTC) concentration

**Fig. 6:**
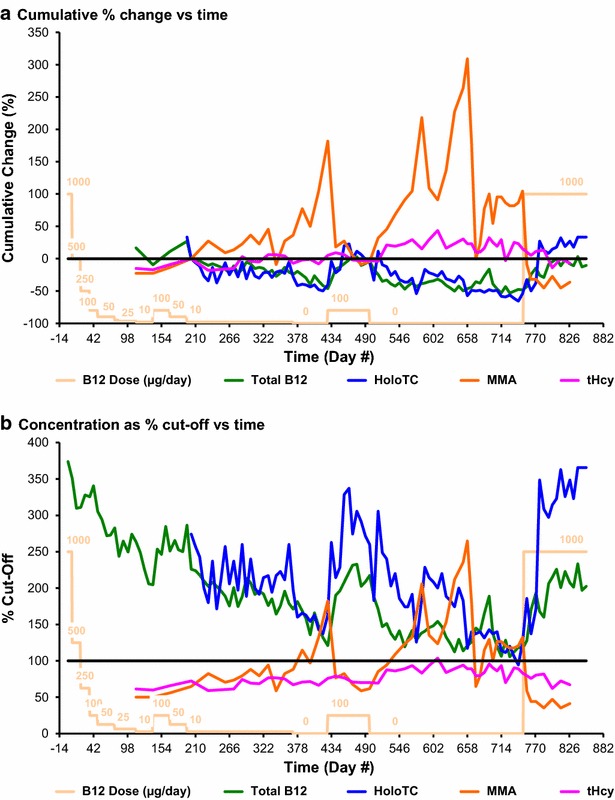
Cumulative change and cut-off values. In **a**, for each measurement for each analyte, cumulative change (%) = 100 × [(initial value − current value)/initial value]. In **b**, for each measurement for each analyte, % cut-off value = 100 × (measured value/laboratory cut-off limit value)

**Fig. 7:**
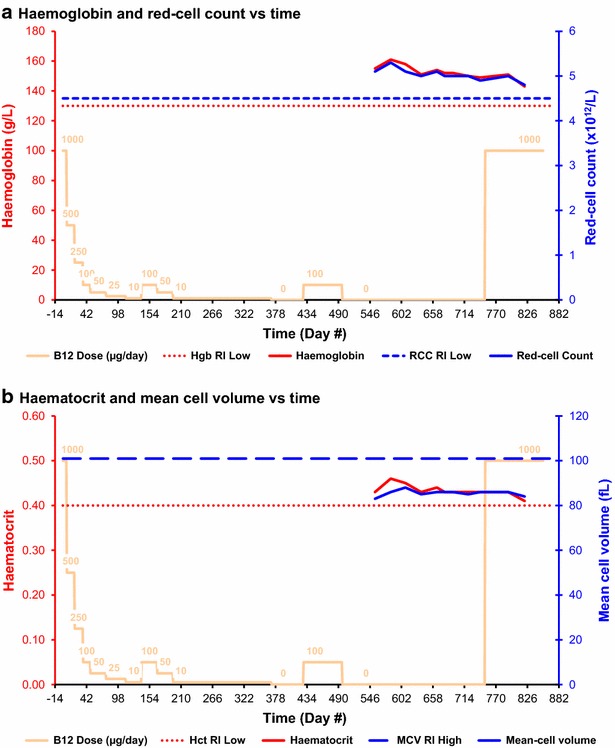
Haematology. In **a**, Hgb low = low limit for haemoglobin concentration defined by Bates and Lewis (2012); RCC low = low limit for red-cell count defined by Bates and Lewis (2012). In **b**, Hct low = low limit for haematocrit defined by Bates and Lewis (2012); MCV high = upper limit for mean cell volume defined by Bates and Lewis (2012)

### Variation over time: vitamin B_12_ immunoassays

#### Serum total vitamin B_12_ immunoassay

The serum total vitamin B_12_ concentration (Fig. 1a) was initially 606 pmol/L, well above the lower limit of the laboratory reference interval of 162 pmol/L, the Bates and Lewis (2012) minimum of 132 pmol/L and the Oh and Brown (2003) minimum of 295 pmol/L, indicating that the subject was replete in serum vitamin B_12_. The concentration then fell as the oral cyanocobalamin supplement dose was reduced from 1000 μg/day, and rose again during the two short periods when the supplementation was increased to 100 μg/day. On day 728, 231 days after the supplementation was finally reduced to 0 μg/day, the total serum vitamin B_12_ concentration fell to a minimum of 171 pmol/L; this was slightly above the lower limit of the laboratory reference interval, and significantly above the Bates and Lewis minimum, but significantly below the Oh and Brown minimum.

#### Serum holotranscobalamin (HoloTC) immunoassay

Results for HoloTC (Figs. 1a, 2a, 3a) were not available until day 196 because of unexpected delays in the initial setting up for this test. The HoloTC concentration on day 196 was reported as >128 pmol/L, above the maximum assay limit, indicating that the subject was replete in serum vitamin B_12_. The concentration fell as the oral cyanocobalamin supplement level was reduced, then varied within a range of 60–91 pmol/L after the oral cyanocobalamin supplement dose was held at 10 μg/day. The concentration then fell to a first minimum, of 48 pmol/L on day 420, after the after the oral cyanocobalamin supplement dose was reduced to 0 μg/day. The concentration then rose to a peak, of 118 pmol/L on day 463, after the oral cyanocobalamin supplement dose was increased to 100 μg/day. The concentration then inexplicably rose, to a peak of 107 pmol/L on day 511, 14 days after the cyanocobalamin supplement dose was finally reduced to 0 μg/day. There were other unexplained variations in HoloTC concentration, over a range of 41–80 pmol/L, before it fell to a minimum of 33 pmol/L on day 742; this was the only time that the concentration fell below the lower limit of the laboratory reference interval of 35 pmol/L.

### Variation over time: vitamin B_12_ metabolite assays

#### Plasma methylmalonic acid (MMA)

Plasma MMA concentration (Figs. 2a, 4a) was not tested until day 112 because previous self-experimentation had shown that increases above baseline would not occur until after serum vitamin B_12_ fell below about 300 pmol/L. The MMA concentration was initially 0.17 μmol/L, on day 112, well below the upper limit of the laboratory reference interval of 0.34 μmol/L. The MMA concentration then increased, even during the first of the two short periods when the supplementation was increased to 100 μg/day, and continued to increase as the cyanocobalamin dose was decreased to 10 μg/day. The MMA concentration briefly and inexplicably fell to 0.20 μmol/L, on day 343, after reaching an initial peak of 0.32 μmol/L on day 330. The MMA concentration rose to a peak of 0.62 μmol/L on day 428, well above the upper limit of the laboratory reference interval, 58 days after the cyanocobalamin dose was initially decreased to 0 μg/day. The MMA concentration then fell to 0.20 μmol/L on day 483, during the second of the two short periods when the supplementation was increased to 100 μg/day. After the cyanocobalamin dose was finally decreased to 0 μg/day, the MMA concentration reached another peak, of 0.70 μmol/L on day 483, then inexplicably fell to 0.42 μmol/L on day 609 before reaching a maximum peak of 0.90 μmol/L on day 658. The MMA concentration then again inexplicably fell, to 0.22 μmol/L on day 672, before increasing to remain within a range of 0.34–0.45 μmol/L, until the cyanocobalamin dose was increased to 1000 μg/day, then it finally fell to 0.12 μmol/L.

#### Plasma total homocysteine (tHcy)

As for the MMA, plasma tHcy concentration (Fig. 3a) was also not tested until day 112 because previous self-experimentation had shown that increases above baseline would not occur until after serum vitamin B_12_ fell below about 300 pmol/L. The tHcy concentration was initially 8.4 μmol/L, on day 112, well below the upper limit of the laboratory reference interval of 13.7 μmol/L. The tHcy concentration slowly increased, without any definite effect of the two short periods when the supplementation was increased to 100 μg/day. The tHcy concentration reached a maximum of 14.2 μmol/L, on day 609, 112 days after the cyanocobalamin dose was finally decreased to 0 μg/day. The tHcy concentration then inexplicably gradually fell, reaching 11.4 μmol/L before the cyanocobalamin dose was increased to 1000 μg/day, then finally fell to 8.5 μmol/L.

### Variation over time: derived data

#### Holotranscobalamin (HoloTC) as percentage of total serum vitamin B_12_

The HoloTC concentration, calculated as a percentage of the total serum vitamin B_12_ concentration (Fig. 5a), was initially 27.6 %, on day 196. The percentage HoloTC varied significantly, over a range of 16.3–38.6 % during the vitamin B_12_ depletion stage. During this time there were several distinct peaks and troughs, but these were not all clearly related to any change in the cyanocobalamin dose. One peak, of 37.6 % on day 434, occurred 6 days after commencement of the second of the two short periods when the supplementation was increased to 100 μg/day, and one trough, of 21.1 % occurred 7 days after the end of that period. Another inexplicable peak, of 35.1 %, occurred after the cyanocobalamin dose was finally decreased to 0 μg/day. The percentage HoloTC reached a maximum of 44.9 % on day 777, 26 days after the cyanocobalamin dose was increased to 1000 μg/day.

#### Holohaptocorrin (HoloHC)

The HoloHC concentration, calculated by subtracting the HoloTC concentration from the total vitamin B_12_ (Fig. 5b), was initially 336 pmol/L, on day 196. The HoloHC concentration fell to 139 pmol/L on day 428, before the second of the two short periods when the supplementation was increased to 100 μg/day, rose to a peak of 279 pmol/L on day 469 during that period, then fell to 151 pmol/L on day 518 after the cyanocobalamin dose was finally decreased to 0 μg/day. There were then two inexplicable peaks, the second reaching 256 pmol/L on day 694, while the cyanocobalamin dose remained at 0 μg/day. The HoloHC concentration increased rapidly, from 142 to 253 pmol/L, after the cyanocobalamin dose was increased to 1000 μg/day, then fell inexplicably to 150 pmol/L before rapidly rising again.

### Comparisons of analyte concentration variation over time: vitamin B_12_ and metabolite assays

#### Cumulative change in analyte concentration as percentage of initial value

The cumulative changes in total vitamin B_12_, HoloTC, MMA and tHcy over time are shown in Fig. 6a. Day 203 was chosen as the reference point for the initial values because it was the first day on which concurrent values were obtained for all analytes. Plasma MMA was the most sensitive indicator of vitamin B_12_ deficiency, increasing by a maximum of 309 % above the initial value on day 658 but, as noted above, there were unexplained anomalies. Plasma tHcy increased by a maximum of 43 % above the initial value on day 609 and, as noted above, was only this once above the upper limit of the laboratory reference interval value. Total vitamin B_12_ fell by a maximum of 53 % below the initial value on day 728, and HoloTC fell by a maximum of 66 % below the initial value on day 742.

#### Analyte concentration as percentage of cut-off value

The analyte concentrations, as a percentage of the quoted cut-off values, of total vitamin B_12_, HoloTC, MMA and tHcy over time are shown in Fig. 6b. Plasma MMA was the most sensitive indicator of vitamin B_12_ deficiency, increasing to a maximum of 265 % of the cut-off value on day 658 but, as noted above, there were unexplained anomalies. Plasma tHcy increased to a maximum of 104 % of the cut-off value on day 609 and, as noted above, was only this once above the upper limit of the laboratory reference interval value. Total vitamin B_12_ fell to a minimum of 106 % of the cut-off value on day 728, and HoloTC fell to a minimum of 89 % of the cut-off value on day 742.

### Variation over time: haematology

#### Haemoglobin and red-cell count

Haemoglobin and red-cell count were not monitored until day 555 because previous self-experimentation had shown that this subject’s haematology was unaffected by this level of vitamin B_12_ deficiency. The initial haemoglobin and red-cell count were 155 g/L and 5.1 × 10^12^/L respectively, both well within the reference normal ranges (Fig. 7a). Both fell slightly and insignificantly, to 149 g/L and 4.9 × 10^12^/L respectively, by the end of the vitamin B_12_ depletion stage. Both haemoglobin and red-cell count rose and then fell slightly and insignificantly after the cyanocobalamin dose was increased to 1000 μg/day, with final values of 143 g/L and 4.8 × 10^12^/L respectively. The overall trend for haemoglobin and red-cell count was a slight fall, of 8 and 6 % respectively, not significantly affected by the final change in cyanocobalamin dose from 0 to 1000 μg/day.

#### Haematocrit and mean-cell volume

The initial haematocrit and mean-cell volume were 0.43 (or 43 %) and 83 fL respectively, on day 555, both well within the reference normal ranges (Fig. 7b). There was no significant change in either analyte during the remainder of the vitamin B_12_ depletion stage, and neither was significantly affected by the final change in cyanocobalamin dose from 0 to 1000 μg/day.

### Analyte concentration correlations and significance: vitamin B_12_ and metabolite assays

Calculations of correlation and significance were performed using Microsoft Excel; complete details may be found in table T3 of Additional file 1: Table S1. Charts for analyte concentration correlations are shown in Figs. 1b, 2b, 3b and 4b. Results for Pearson’s correlation coefficient (r) and probability (p) are summarised in Table 1.

**Table 1:** Results for correlation coefficient (r) and p value (p)

Figure	Analytes	N	r	p
1b	Total B_12_ and HoloTC	95	0.70	2.9 × 10^−15^
2b	MMA and HoloTC	52	−0.52	7.6 × 10^−5^
3b	tHcy and HoloTC	52	−0.45	9.2 × 10^−4^
4b	Total B_12_ and MMA	55	−0.67	2.9 × 10^−8^

N = number of concurrent test results for each analyte pair, r = Pearson’s correlation coefficient between analyte concentrations, p = p value = probability of obtaining the result ≥r, for N concurrent test results, if there is no actual linear correlation between analyte concentrations and that the apparent correlation is due to random chance

#### Total serum vitamin B_12_ versus holotranscobalamin (HoloTC)

There was a very highly significant positive correlation, with r = 0.70), between total serum vitamin B_12_ and HoloTC concentrations (Fig. 1b; Table 1). This result is consistent with no great difference in sensitivity of the total serum vitamin B_12_, compared to HoloTC, to the onset of vitamin B_12_ deficiency, but does not tell us which the most sensitive indicator is. This result is also consistent with the absence of any remarkable differences in response over time.

#### Plasma methylmalonic acid (MMA) versus holotranscobalamin (HoloTC)

The negative correlation between MMA and HoloTC concentrations was moderate, with r = −0.52 (Fig. 2b; Table 1). This result is consistent with the observed difference in sensitivity of the MMA, compared to HoloTC, to the onset of vitamin B_12_ deficiency but does not itself tell us which the most sensitive indicator is.

#### Plasma total homocysteine (tHcy) versus holotranscobalamin (HoloTC)

The correlation between tHcy and HoloTC concentrations was weak to moderate, with r = 0.45 (Fig. 3b; Table 1). This result is also consistent with the low sensitivity of both the HoloTC and tHcy to the onset of vitamin B_12_ deficiency in this subject.

#### Total serum vitamin B_12_ versus plasma methylmalonic acid (MMA)

There was a very highly significant negative correlation, with r = −0.67, between MMA and total serum vitamin B_12_ concentrations (Fig. 4b; Table 1). This result could be consistent with no great difference in sensitivity of the MMA, compared to serum vitamin B_12_, to the onset of vitamin B_12_ deficiency, but is not inconsistent with the observed higher sensitivity of the MMA to the onset of vitamin B_12_ deficiency.

### Analyte sensitivity comparisons: vitamin B_12_ and metabolite assays

Analyte sensitivity comparisons were performed, using Microsoft Excel, by comparing the number of concurrent pairs of results in each chart quadrant; complete details may be found in table T3 of Additional file 1: Table S1. Results for counts, for the number of results for each analyte pair within each chart quadrant for charts shown in Figs. 1b, 2b, 3b and 4b, are summarised in Table 2.

**Table 2:** Calculated counts for analyte sensitivity comparisons

Figure	Count criteria	Reference	Quadrant	Count
1b	Concurrent B_12_ and HoloTC tests (N)			95
1b	B_12_ ≥162 pmol/L and HoloTC ≥35 pmol/L	L	1	94
1b	B_12_ ≥295 pmol/L and HoloTC ≥35 pmol/L	A	1	41
1b	B_12_ ≥133 pmol/L and HoloTC ≥35 pmol/L	B	1	94
1b	B_12_ ≥162 pmol/L and HoloTC <35 pmol/L	L	2	1
1b	B_12_ ≥295 pmol/L and HoloTC <35 pmol/L	A	2	0
1b	B_12_ ≥133 pmol/L and HoloTC <35 pmol/L	B	2	1
1b	B_12_ <162 pmol/L and HoloTC <35 pmol/L	L	3	0
1b	B_12_ <295 pmol/L and HoloTC <35 pmol/L	A	3	1
1b	B_12_ <133 pmol/L and HoloTC <35 pmol/L	B	3	0
1b	B_12_ <162 pmol/L and HoloTC ≥35 pmol/L	L	4	0
1b	B_12_ <295 pmol/L and HoloTC ≥35 pmol/L	A	4	53
1b	B_12_ <133 pmol/L and HoloTC ≥35 pmol/L	B	4	0
2b	Concurrent MMA and HoloTC Tests (N)			52
2b	MMA ≥0.34 µmol/L and HoloTC ≥35 pmol/L	L	1	23
2b	MMA ≥0.34 µmol/L and HoloTC <35 pmol/L	L	2	1
2b	MMA <0.34 µmol/L and HoloTC <35 pmol/L	L	3	0
2b	MMA <0.34 µmol/L and HoloTC ≥35 pmol/L	L	4	28
3b	Concurrent tHcy and HoloTC tests (N)			52
3b	tHcy ≥13.7 µmol/L and HoloTC ≥35 pmol/L	L	1	1
3b	tHcy ≥13.7 µmol/L and HoloTC <35 pmol/L	L	2	0
3b	tHcy <13.7 µmol/L and HoloTC <35 pmol/L	L	3	1
3b	tHcy <13.7 µmol/L and HoloTC ≥35 pmol/L	L	4	50
4b	Concurrent MMA and B_12_ tests (N)			55
4b	MMA ≥0.34 µmol/L and B_12_ ≥162 pmol/L	L	1	24
4b	MMA ≥0.34 µmol/L and B_12_ ≥295 pmol/L	A	1	1
4b	MMA ≥0.34 µmol/L and B_12_ ≥133 pmol/L	B	1	24
4b	MMA <0.34 µmol/L and B_12_ ≥162 pmol/L	L	2	31
4b	MMA <0.34 µmol/L and B_12_ ≥295 pmol/L	A	2	20
4b	MMA <0.34 µmol/L and B_12_ ≥133 pmol/L	B	2	31
4b	MMA <0.34 µmol/L and B_12_ <162 pmol/L	L	3	0
4b	MMA <0.34 µmol/L and B_12_ <295 pmol/L	A	3	11
4b	MMA <0.34 µmol/L and B_12_ <133 pmol/L	B	3	0
4b	MMA ≥0.34 µmol/L and B_12_ <162 pmol/L	L	4	0
4b	MMA ≥0.34 µmol/L and B_12_ <295 pmol/L	A	4	23
4b	MMA ≥0.34 µmol/L and B_12_ <133 pmol/L	B	4	0

Reference L = analyte concentration limit defined by the testing laboratory, reference A = analyte concentration limit defined by Oh and Brown (2003), reference B = analyte concentration limit defined by Bates and Lewis (2012), Quadrant = chart quadrant: , Count = number of concurrent analyte pair concentration results within each quadrant

#### Total serum vitamin B_12_ versus holotranscobalamin (HoloTC)

The low sensitivity of the HoloTC and total serum vitamin B_12_ concentrations to the onset of vitamin B_12_ deficiency are illustrated in Fig. 1b and Table 2. Of the 95 concurrent test results, the 94 data points in the first quadrant show serum vitamin B_12_ above the lower limit of the laboratory reference interval of 162 pmol/L (indicating vitamin B_12_ adequacy) while HoloTC also is above the lower limit of the laboratory reference interval of 35 pmol/L (also indicating vitamin B_12_ adequacy). The one remaining data point is in the second quadrant, showing HoloTC below the lower limit of the laboratory reference interval (indicating vitamin B_12_ deficiency) while total serum vitamin B_12_ is above the lower limit of the laboratory reference interval (indicating vitamin B_12_ adequacy).

The interpretation of this chart changes significantly if reference levels for total serum vitamin B_12_ concentrations from Oh and Brown (2003) are used instead of those quoted by the testing laboratory. By increasing the minimum total serum vitamin B_12_ concentration from the laboratory reference level of 162–295 pmol/L (Oh and Brown 2003), many data points move from the first to the fourth quadrant. Using the Bates and Lewis (2012) reference level of 133 pmol/L does not move any data points from the first to the fourth quadrant.

In the case of the Oh and Brown reference level, of the 95 concurrent test results, only the 41 data points now in the first quadrant show serum vitamin B_12_ above the Oh and Brown reference minimum of 295 pmol/L (indicating vitamin B_12_ adequacy) while HoloTC also is above the lower limit of the laboratory reference interval of 35 pmol/L (also indicating vitamin B_12_ adequacy). There are now 53 data points in the fourth quadrant, showing HoloTC above the lower limit of the laboratory reference interval (indicating vitamin B_12_ adequacy) while total serum vitamin B_12_ is below the Oh and Brown reference minimum (indicating vitamin B_12_ deficiency).

#### Plasma methylmalonic acid (MMA) versus holotranscobalamin (HoloTC)

The low sensitivity of the HoloTC concentration to the onset of vitamin B_12_ deficiency, compared to MMA, is illustrated in Fig. 2b and Table 2. Of the 52 concurrent test results, the 23 data points in the first quadrant show MMA above the upper limit of the laboratory reference interval of 0.34 µmol/L (indicating vitamin B_12_ deficiency) while HoloTC is above the lower limit of the laboratory reference interval of 35 pmol/L (indicating vitamin B_12_ adequacy). Only one data point is in the second quadrant, showing MMA above the upper limit of the laboratory reference interval while HoloTC is below the lower limit of the laboratory reference interval (both indicating vitamin B_12_ deficiency). The remaining 28 data points are in the fourth quadrant, showing MMA below the upper limit of the laboratory reference interval while HoloTC is above the lower limit of the laboratory reference interval (both indicating vitamin B_12_ adequacy).

#### Plasma total homocysteine (tHcy) versus holotranscobalamin (HoloTC)

The low sensitivity of the HoloTC and tHcy concentrations to the onset of vitamin B_12_ deficiency are illustrated in Fig. 3b and Table 2. Of the 52 concurrent test results, the one data point in the first quadrant shows tHcy above the upper limit of the laboratory reference interval of 13.7 μmol/L (indicating vitamin B_12_ deficiency) while HoloTC is above the lower limit of the laboratory reference interval of 35 pmol/L (indicating vitamin B_12_ adequacy). One data point is in the third quadrant, showing tHcy below the upper limit of the laboratory reference interval (indicating vitamin B_12_ adequacy) while HoloTC is above the lower limit of the laboratory reference interval (indicating vitamin B_12_ deficiency). The remaining 50 data points are in the fourth quadrant, showing tHcy below the upper limit of the laboratory reference interval while HoloTC is above the lower limit of the laboratory reference interval (both indicating vitamin B_12_ adequacy).

#### Total serum vitamin B_12_ versus plasma methylmalonic acid (MMA)

The high sensitivity of the MMA and low sensitivity of total serum vitamin B_12_ concentrations to the onset of vitamin B_12_ deficiency are illustrated in Fig. 4b and Table 2. Of the 55 concurrent test results, the 24 data points in the first quadrant show MMA above the upper limit of the laboratory reference interval 0.34 µmol/L (indicating vitamin B_12_ deficiency) while total serum vitamin B_12_ is above the lower limit of the laboratory reference interval of 162 pmol/L (indicating vitamin B_12_ adequacy). The remaining 31 data points are in the second quadrant, showing MMA below the upper limit of the laboratory reference interval (indicating vitamin B_12_ adequacy) while total serum vitamin B_12_ also is above the lower limit of the laboratory reference interval (also indicating vitamin B_12_ adequacy).

As with the previous chart, the interpretation changes significantly if reference levels for total serum vitamin B_12_ concentrations from Oh and Brown (2003) are used instead of those quoted by the testing laboratory. By increasing the minimum total serum vitamin B_12_ concentration from the laboratory reference level of 162–295 pmol/L (Oh and Brown 2003), many data points move from the first and second quadrants to the fourth and third quadrants respectively. Using the Bates and Lewis (2012) reference level of 180 pmol/L does not move any data points between quadrants.

In the case of the Oh and Brown reference level, the single data point remaining in the first quadrant shows MMA above the upper limit of the laboratory reference interval 0.34 µmol/L (indicating vitamin B_12_ deficiency) while total serum vitamin B_12_ is above the lower limit of the laboratory reference interval of (indicating vitamin B_12_ adequacy). There are now 20 data points in the second quadrant, showing MMA below the upper limit of the laboratory reference interval (indicating vitamin B_12_ adequacy) while total serum vitamin B_12_ is above the Oh and Brown reference minimum (also indicating vitamin B_12_ adequacy). There are now 11 data points in the third quadrant, showing MMA below the upper limit of the laboratory reference interval (indicating vitamin B_12_ adequacy) while total serum vitamin B_12_ also is below the Oh and Brown reference minimum (indicating vitamin B_12_ deficiency). The remaining 23 data points are now in the fourth quadrant, showing MMA above the upper limit of the laboratory reference interval (indicating vitamin B_12_ deficiency) while total serum vitamin B_12_ also is below the Oh and Brown reference minimum (also indicating vitamin B_12_ deficiency).

### The subject

The subject’s weight did not change significantly during the course of the experiment. He observed worsening neurological symptoms including extreme mental tiredness, and unpleasant micro-dreams in the daytime. The subject also observed significant reduction in tactile sense in his extremities (peripheral neuropathy), and severe physical fatigue. Recovery following resumption of oral vitamin B_12_ treatment was very slow.

## Discussion

### Experimental findings

#### Serum total vitamin B_12_ versus serum holotranscobalamin (HoloTC)

Three graphical methods have shown that serum HoloTC was only slightly more sensitive to the onset of vitamin B_12_ deficiency than serum total vitamin B_12_, with HoloTC indicating vitamin B_12_ deficiency and serum total vitamin B_12_ indicating vitamin B_12_ adequacy for only 1 of the 95 concurrent tests. Firstly, there were no remarkable differences in the time charts for the two analyte concentrations (Figs. 1a, 6). Secondly, there was a very highly significant positive correlation between serum total vitamin B_12_ and HoloTC concentrations (Fig. 1b; Table 1), consistent with the similar responses over time. Thirdly, all except one data point were in the first quadrant of the analyte sensitivity comparison chart, indicating that both analytes were within the normal range for 94 of the 95 concurrent tests when using the laboratory reference level serum total vitamin B_12_ (Fig. 1b; Table 2).

It is important to note that the relative sensitivities can be very significantly altered by changing the reference level for either or both analytes. By using the Oh and Brown (2003) reference level (295 pmol/L) for serum total vitamin B_12_, instead of the lower limit of the laboratory reference interval for serum total vitamin B_12_ (162 pmol/L), HoloTC becomes far less sensitive than serum total vitamin B_12_ to the onset of vitamin B_12_ deficiency (Fig. 1a, b).

#### Plasma methylmalonic acid (MMA) versus serum holotranscobalamin (HoloTC)

Three graphical methods have shown that serum HoloTC was far less sensitive to the onset of vitamin B_12_ deficiency than plasma MMA, with serum HoloTC indicating vitamin B_12_ adequacy while MMA indicated vitamin B_12_ deficiency for 23 of the 52 concurrent tests. Firstly, there were very remarkable differences in the time charts for the two analyte concentrations (Figs. 2a, 6). Secondly, although there was a moderate correlation between MMA and HoloTC concentrations (Fig. 2b; Table 1), this is not inconsistent with the different responses over time. Thirdly, only one data point was in the second quadrant of the analyte sensitivity comparison chart, indicating that HoloTC was below the lower limit of the laboratory reference interval of 35 pmol/L only once, whereas 23 of the 52 data points were in the first quadrant, indicating that MMA was above the upper limit of the laboratory reference interval of 0.34 µmol/L while HoloTC was above the lower limit of the laboratory reference interval (Fig. 2b; Table 2).

Although plasma MMA showed the greatest sensitivity to the onset of vitamin B_12_ deficiency, the results included several unexplained anomalies (Figs. 2a, 6).

#### Plasma total homocysteine (tHcy) versus serum holotranscobalamin (HoloTC)

Three graphical methods have shown that serum HoloTC was similarly insensitive to the onset of vitamin B_12_ deficiency as plasma tHcy, with both indicating vitamin B_12_ adequacy for 50 of the 52 concurrent tests. Firstly, there were no remarkable differences in the time charts for the two analyte concentrations except that, as expected, they tended to change in opposite directions (Figs. 3a, 6). Secondly, there was a weak to moderate correlation between tHcy and HoloTC concentrations (Fig. 3b; Table 1), not inconsistent with both having low sensitivity to the onset of vitamin B_12_ deficiency. Thirdly, 50 of the 52 data points were in the fourth quadrant of the analyte sensitivity comparison chart, indicating that HoloTC was above the lower limit of the laboratory reference interval of 35 pmol/L while tHcy was below the upper limit of the laboratory reference interval of 13.7 μmol/L (Fig. 3b; Table 2).

The low sensitivity of the homocysteine is consistent with the absence of any significant haematological affects (Fig. 7a, b). The absence of anaemia does not rule out vitamin B_12_ deficiency; as noted by Lindenbaum et al. (1988), 28 % of patients with neurological affects due to vitamin B_12_ deficiency have no haematological abnormalities.

### Comparison with previous experimental findings

The results of this experiment are inconsistent with the reported findings that HoloTC was the most sensitive and earliest marker of vitamin B_12_ deficiency. Firstly, HoloTC was not significantly more sensitive than total vitamin B_12_ to the depletion of the vitamin B_12_ body store (Figs. 1, 6). Secondly, MMA overtly showed a disturbed metabolism well before HoloTC indicated any vitamin B_12_ deficiency (Figs. 2, 6).

As noted earlier in this discussion, it is possible to alter the apparent relative sensitivity of any pair of analytes by selectively changing the cut-off value of one or both of them. As demonstrated in this experiment, selecting different cut-off values for total vitamin B_12_ changed the relative sensitivities of HoloTC and total vitamin B_12_ (Fig. 1a, b); the same applies to the cut-off value for HoloTC. When the HoloTC cut-off is increased, the sensitivity to vitamin B_12_ deficiency is increased, but the specificity of the test is reduced, increasing the number of false positive results. Conversely, if the HoloTC cut-off is decreased, the specificity of the test is increased, but the sensitivity of the test is reduced, increasing the number of false negative results.

The results of this experiment are consistent with the findings of several researchers whose results do not support the claim that HoloTC is a significantly earlier and more sensitive indicator of vitamin B_12_ deficiency than total vitamin B_12_ and its metabolites (Miller et al. 2005; Clarke et al. 2007; Schrempf et al. 2011; Palacios et al. 2013; Remacha et al. 2014). These studies all involved aged patients whose vitamin B_12_ deficiency was likely to have been caused by food-cobalamin malabsorption (Carmel 1995; Andrès et al. 2004). Miller et al. (2005) concluded that “HoloTC and total vitamin B12 have equal diagnostic accuracy in screening for metabolic vitamin B12 deficiency”. Clarke et al. (2007) reported “modest” superiority of the HoloTC immunoassay, but concluded that neither HoloTC nor total vitamin B_12_ was suitable for screening for vitamin B_12_ deficiency. Schrempf et al. (2011) reported that “holoTC does not show superior diagnostic accuracy compared to VitB12 for the detection of VitB12 deficiency in subjects with neuropsychiatric conditions”. Palacios et al. (2013) found that HoloTC sensitivity was only 44 %, and concluded that it was unsuitable for screening alone. Remacha et al. (2014) concluded that “These data do not support HoloTC as the earliest marker of Cbl deficiency and challenge the classification in stages of Cbl deficiency”.

### Herbert’s model

The results of this experiment are inconsistent with Herbert’s model for *sequential stages in the development of vitamin B*_*12*_*deficiency*, in which HoloTC is the earliest marker of the change from *Normal* to *Early Negative B*_*12*_*Balance* (Herbert 1987a, 1994). Firstly, because the responses of HoloTC and total vitamin B_12_ were so similar, these results do not support the hypothesis that HoloTC is an earlier or more sensitive indicator of a transition between normal and negative balance conditions (Figs. 1, 6). Secondly, as stated above, MMA overtly indicated a disturbed metabolism well before HoloTC indicated any vitamin B_12_ deficiency (Figs. 2, 6).

### Limitations of the experiment

#### Number of subjects

Because this subject has vitamin B_12_ deficiency suspected of being caused by a defect in intracellular metabolism, a relatively rare condition, he might not represent a typical patient. The sensitivity of HoloTC to the onset of a deficiency condition, compared to total vitamin B_12_ and the metabolites, is likely to depend on the specific cause of the deficiency.

For ethical reasons, a longitudinal experiment designed to produce vitamin B_12_ deficiency can only be performed by means of self-experimentation on a single subject. It is therefore not possible to investigate the longitudinal performance of the HoloTC immunoassay for a large group of subjects.

Even if there were no ethical objections to the performance of such an experiment, it would be of limited value. This is because a pure dietary vitamin B_12_ deficiency, induced for the purpose of the experiment, would not necessarily produce the same effect on HoloTC as would other causes of deficiency such as malabsorption. In dietary deficiency, enterohepatic recycling tends to maintain the HoloTC concentration until the liver store of vitamin B_12_ is exhausted; when deficiency develops due to the onset of severe malabsorption, the recycling would become ineffective and produce a faster reduction in HoloTC concentration because it has a very short half-life in serum. (Herbert 1994).

According to Allen B. Weisse (2012), “many self-experiments have proved invaluable to the medical community and to the patients we are seeking to help.” There are numerous other examples of significant contributions made to medical science by single subject experiments (Altman 1998; Widdowson 1993).

#### Number of laboratories

To eliminate individual laboratory error, it would be desirable to have samples tested by more than one laboratory for each analyte. This was not practical because of the high cost, and logistical problems including the need to draw excessive volumes of blood from the subject on each sampling day. For the vitamin B_12_ and HoloTC tests, with modern automated immunoassay systems using pre-packaged reagent kits, the likelihood of significant individual laboratory error has been reduced. For the metabolites, MMA and homocysteine, the specialised laboratory had very strict quality-control procedures. Taking these factors into account, and the very large number of sampling days over such a long time span, it is unlikely that the results can be explained by individual laboratory error.

## Conclusions

The results of this experiment are inconsistent with Herbert’s hypothesis that HoloTC is the earliest marker of vitamin B_12_ deficiency, and therefore do not support his model for the staged development of vitamin B_12_ deficiency. MMA was the most sensitive indicator of vitamin B_12_ deficiency but results contained significant unexplained anomalies. Self-experimentation has produced a detailed record of the response of HoloTC to experimental vitamin B_12_ deficiency, whereas using patients or healthy volunteers as subjects would be unethical.

## Additional files

10.1186/s40064-016-1740-5 Experimental Vitamin B12 Deficiency - for Figures 1 to 7, tables and charts.
10.1186/s40064-016-1740-5 Figures 1 to 7, High-resolution images.
10.1186/s40064-016-1740-5 Figures 1 to 7, High-resolution slides.
