# Supplementary material for: Experimental vitamin B12 deficiency in a human subject: a longitudinal investigation of the performance of the holotranscobalamin (HoloTC, Active-B12) immunoassay
Source: Springerplus. 2016 Feb 25;5:184. doi: 10.1186/s40064-016-1740-5 (PMC4767712; doi:10.1186/s40064-016-1740-5)
Supplement: Supplementary file 2 — 10.1186/s40064-016-1740-5 Figures 1 to 7, High-resolution images. [file 40064_2016_1740_MOESM2_ESM.pdf]

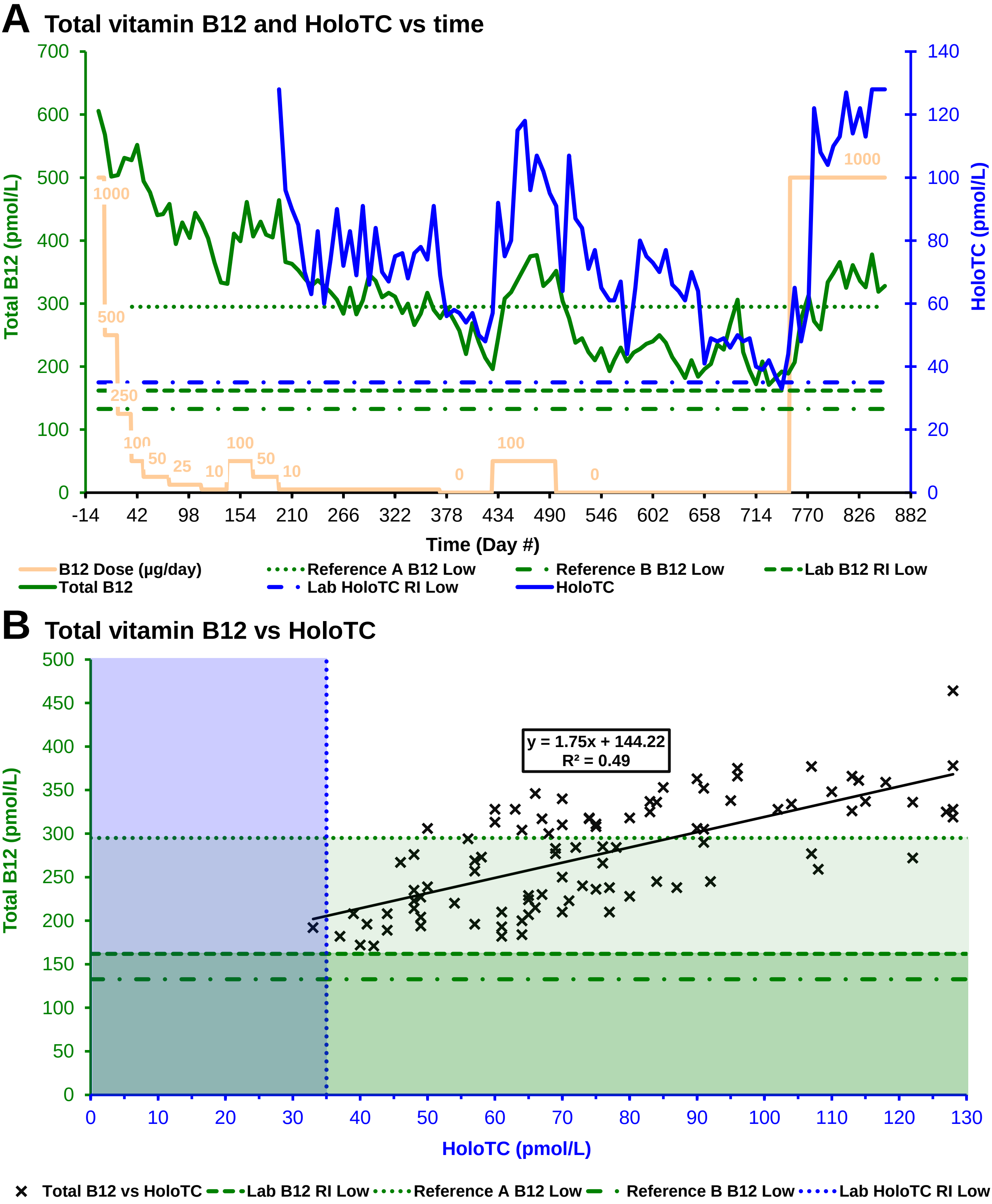

A
Total vitamin B12 and HoloTC vs time
Total B12 (pmol/L)
HoloTC (pmol/L)
Time (Day #)
1000
500
250
100
50
25
10
100
50
10
0
100
0
1000
B12 Dose (µg/day)
Reference A B12 Low
Reference B B12 Low
Lab B12 RI Low
Total B12
Lab HoloTC RI Low
HoloTC
B
Total vitamin B12 vs HoloTC
Total B12 (pmol/L)
HoloTC (pmol/L)
y = 1.75x + 144.22
R² = 0.49
Total B12 vs HoloTC
Lab B12 RI Low
Reference A B12 Low
Reference B B12 Low
Lab HoloTC RI Low

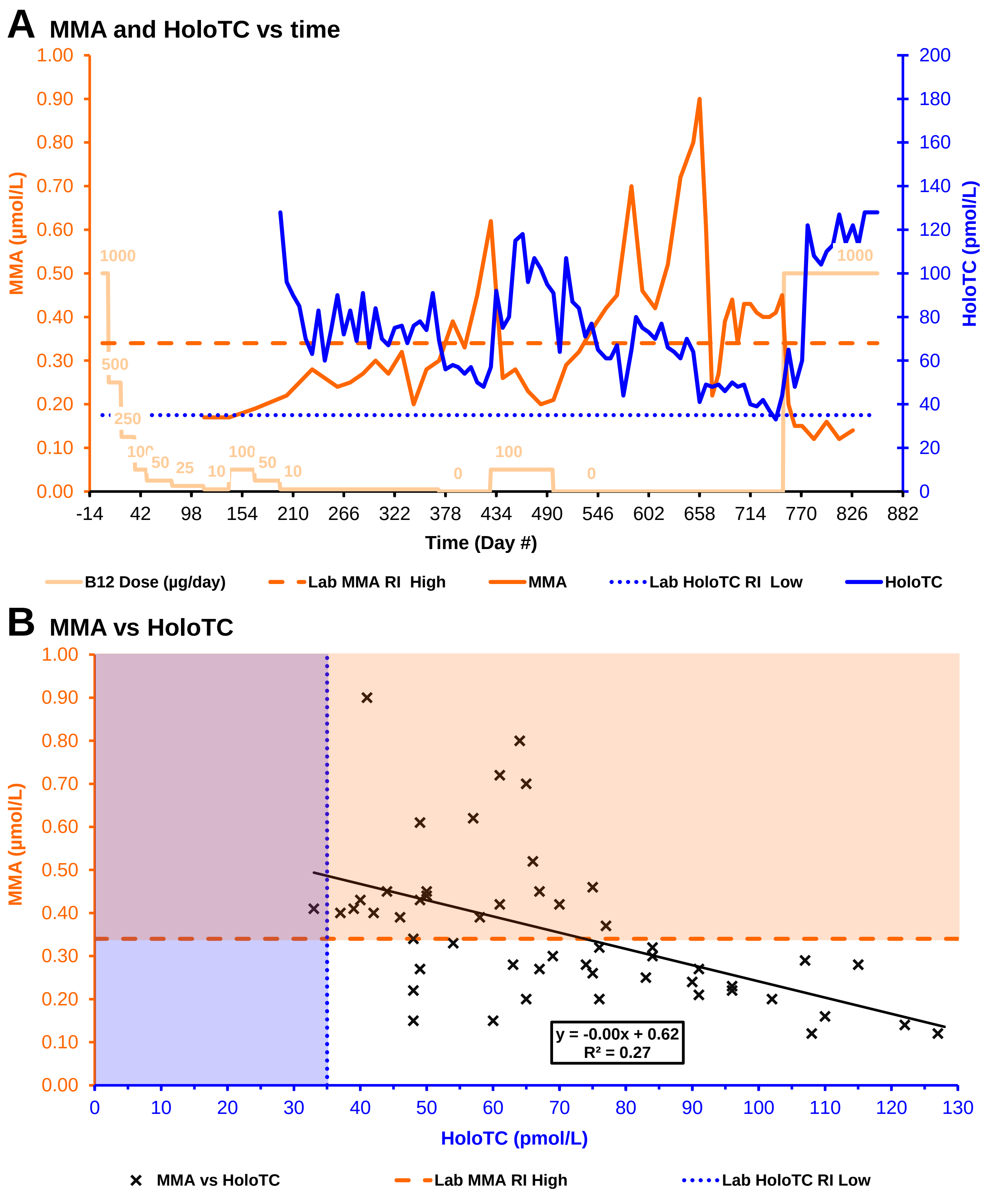
A
MMA and HoloTC vs time
MMA (µmol/L)
HoloTC (pmol/L)
1.00
0.90
0.80
0.70
0.60
0.50
0.40
0.30
0.20
0.10
0.00
200
180
160
140
120
100
80
60
40
20
0
1000
500
250
100
50
25
10
100
50
10
0
100
0
1000
-14
42
98
154
210
266
322
378
434
490
546
602
658
714
770
826
882
Time (Day #)
B12 Dose (µg/day)
Lab MMA RI High
MMA
Lab HoloTC RI Low
HoloTC
B
MMA vs HoloTC
MMA (µmol/L)
1.00
0.90
0.80
0.70
0.60
0.50
0.40
0.30
0.20
0.10
0.00
y = -0.00x + 0.62
R² = 0.27
0
10
20
30
40
50
60
70
80
90
100
110
120
130
HoloTC (pmol/L)
MMA vs HoloTC
Lab MMA RI High
Lab HoloTC RI Low

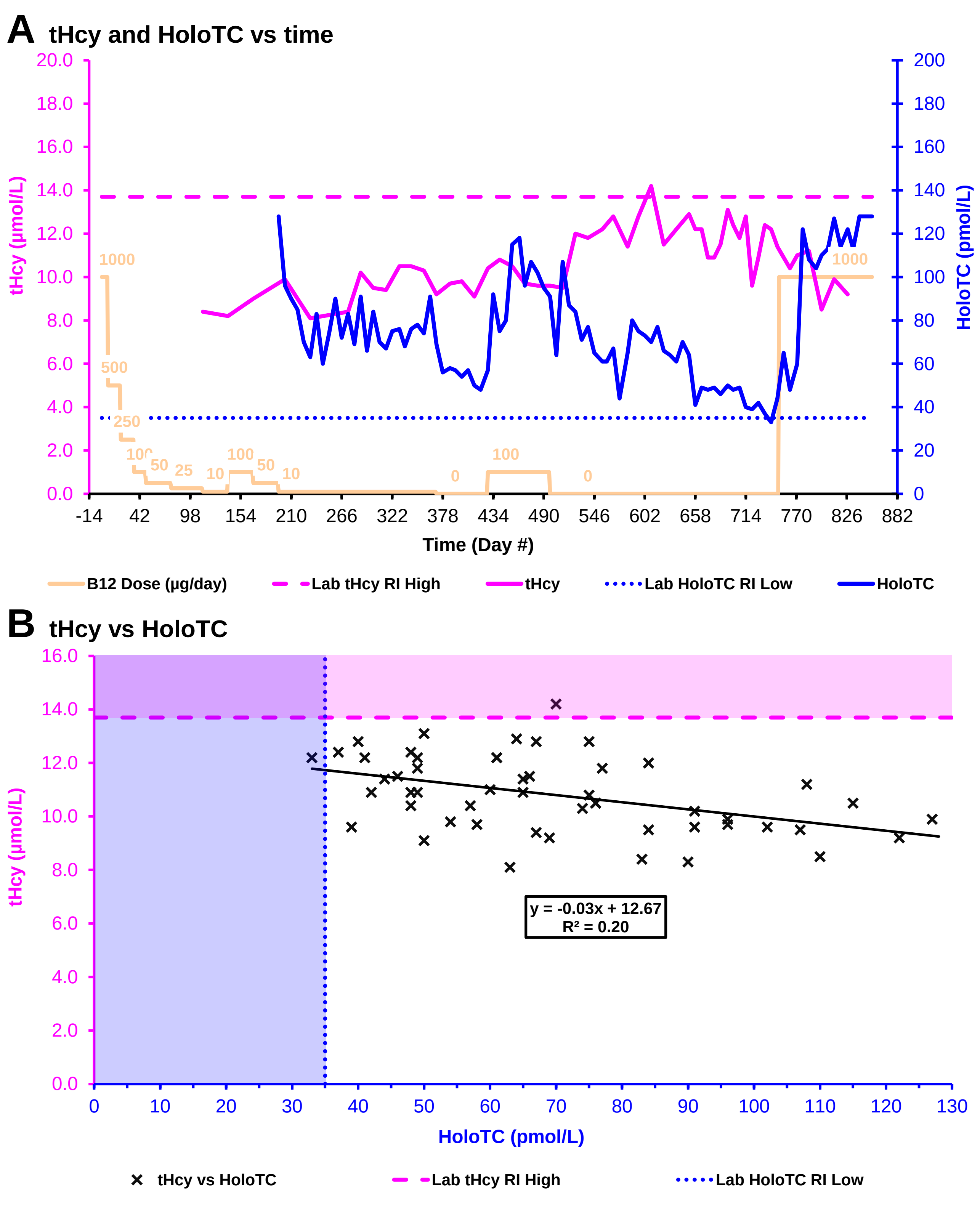
A tHcy and HoloTC vs time
tHcy (µmol/L)
HoloTC (pmol/L)
Time (Day #)
1000
500
250
100
50
25
10
100
50
10
0
100
0
1000
B12 Dose (µg/day)
Lab tHcy RI High
tHcy
Lab HoloTC RI Low
HoloTC
B tHcy vs HoloTC
tHcy (µmol/L)
HoloTC (pmol/L)
y = -0.03x + 12.67
R² = 0.20
tHcy vs HoloTC
Lab tHcy RI High
Lab HoloTC RI Low

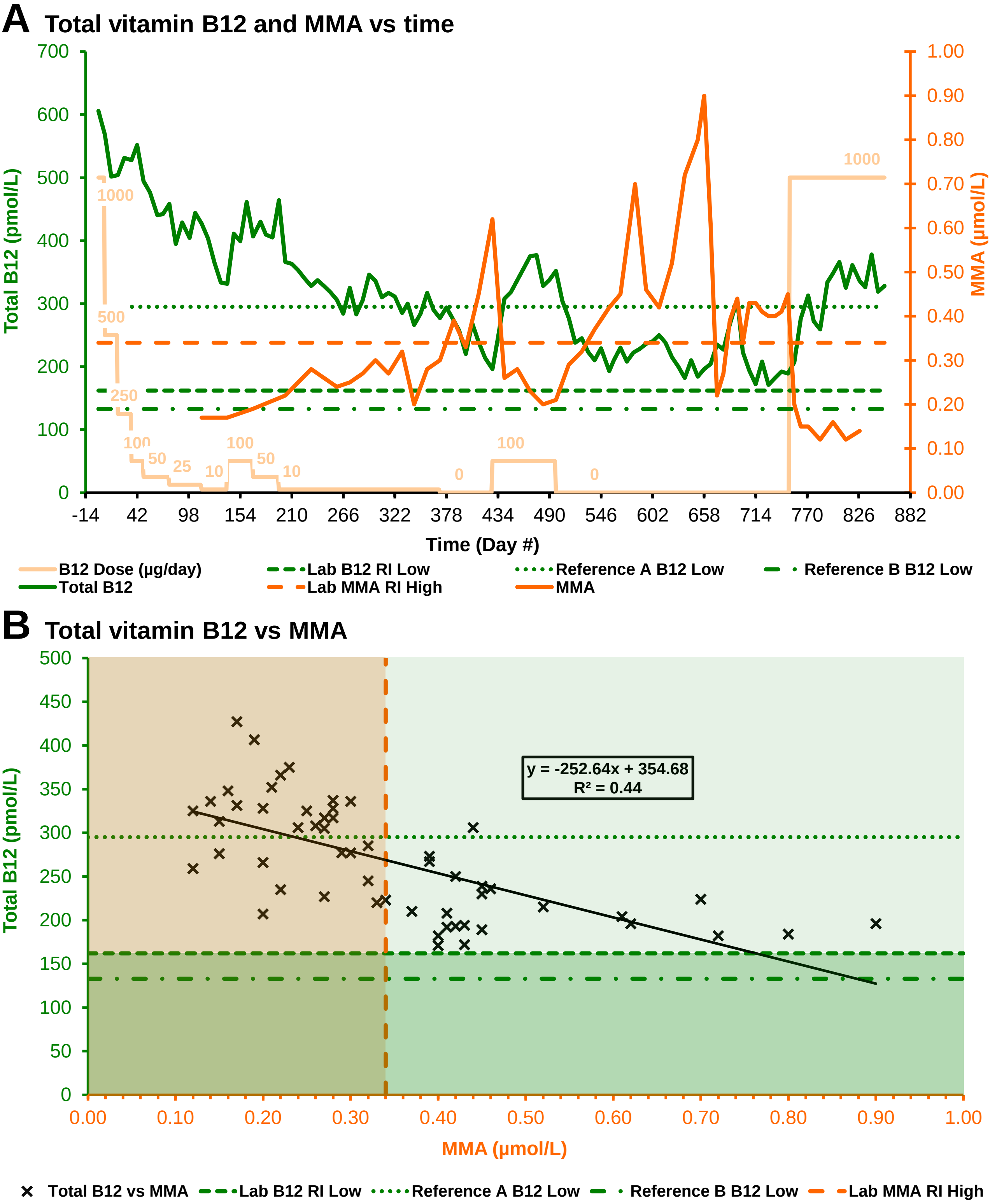
A
Total vitamin B12 and MMA vs time
Total B12 (pmol/L)
MMA (μmol/L)
Time (Day #)
0
100
200
300
400
500
600
700
0.00
0.10
0.20
0.30
0.40
0.50
0.60
0.70
0.80
0.90
1.00
-14
42
98
154
210
266
322
378
434
490
546
602
658
714
770
826
882
1000
500
250
100
50
25
10
100
50
10
0
100
0
1000
B12 Dose (µg/day)
Total B12
Lab B12 RI Low
Lab MMA RI High
Reference A B12 Low
MMA
Reference B B12 Low
B
Total vitamin B12 vs MMA
Total B12 (pmol/L)
MMA (μmol/L)
0
50
100
150
200
250
300
350
400
450
500
0.00
0.10
0.20
0.30
0.40
0.50
0.60
0.70
0.80
0.90
1.00
y = -252.64x + 354.68
R² = 0.44
Total B12 vs MMA
Lab B12 RI Low
Reference A B12 Low
Reference B B12 Low
Lab MMA RI High

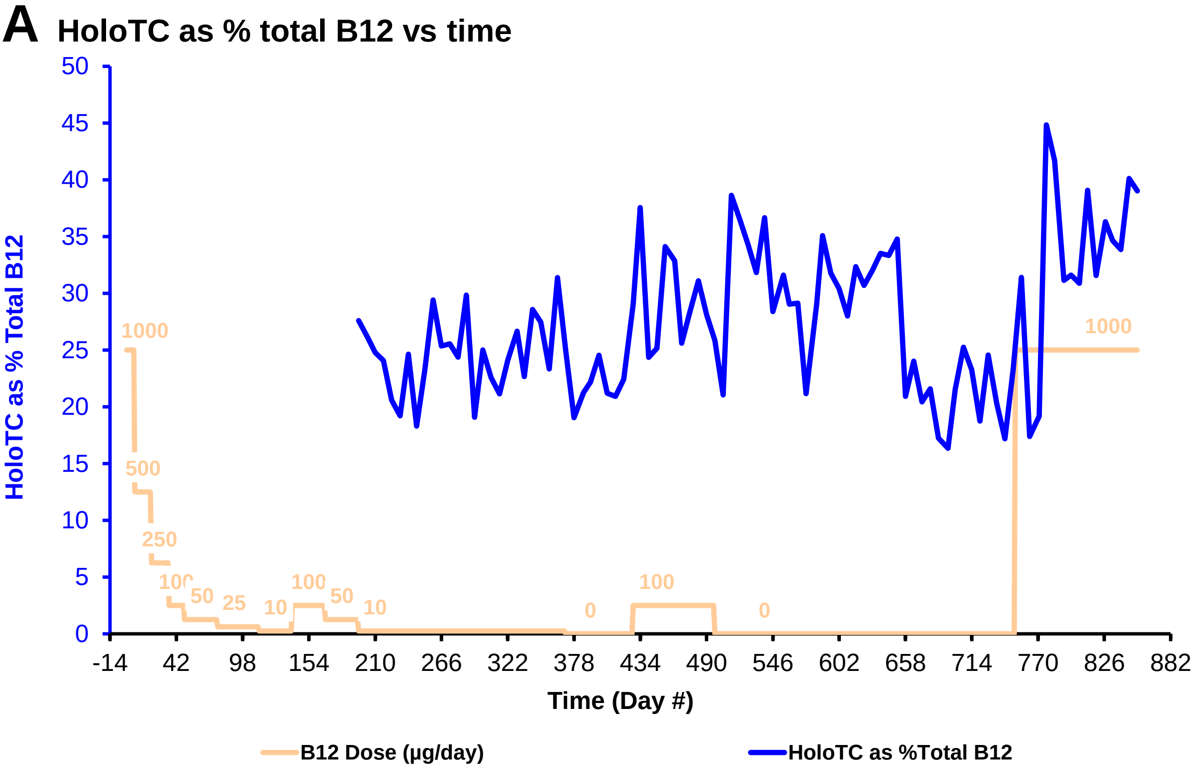
A
HoloTC as % total B12 vs time
HoloTC as % Total B12
Time (Day #)
1000
500
250
100
50
25
10
100
50
10
0
100
0
1000
B12 Dose (µg/day)
HoloTC as %Total B12

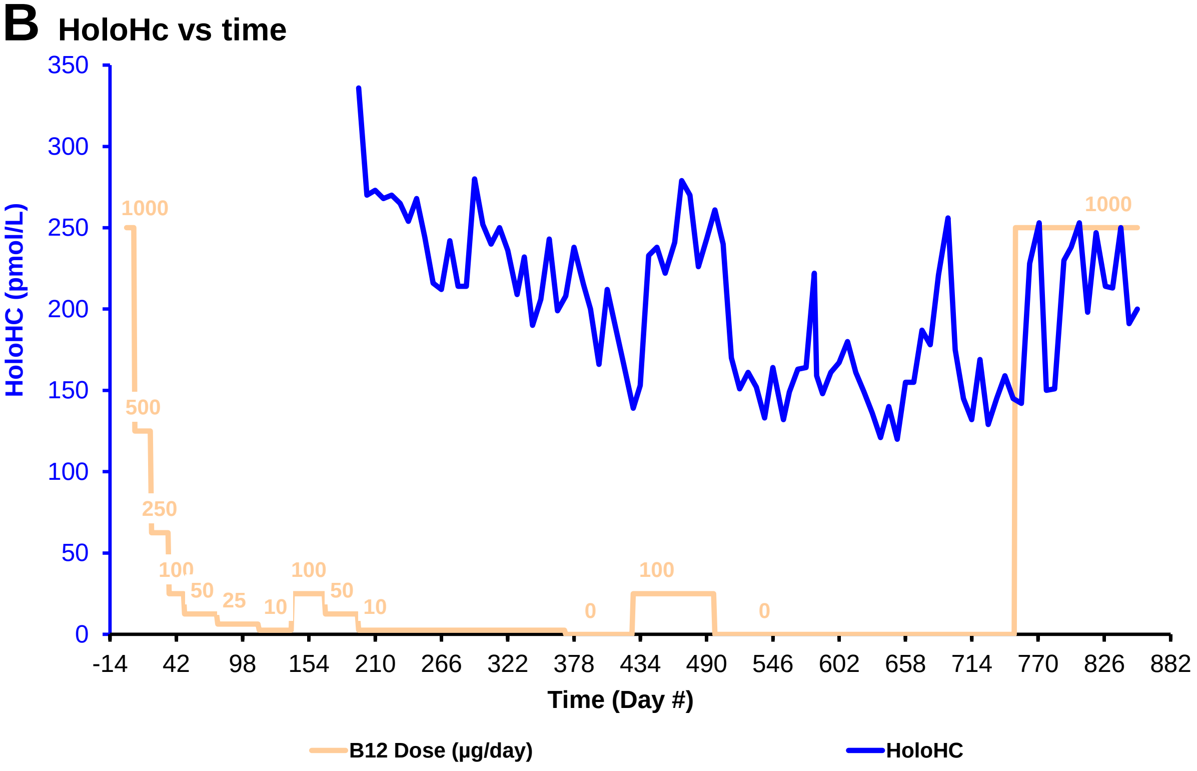
B
HoloHc vs time
HoloHC (pmol/L)
Time (Day #)
1000
500
250
100
50
25
10
100
50
10
0
100
0
1000
B12 Dose (µg/day)
HoloHC

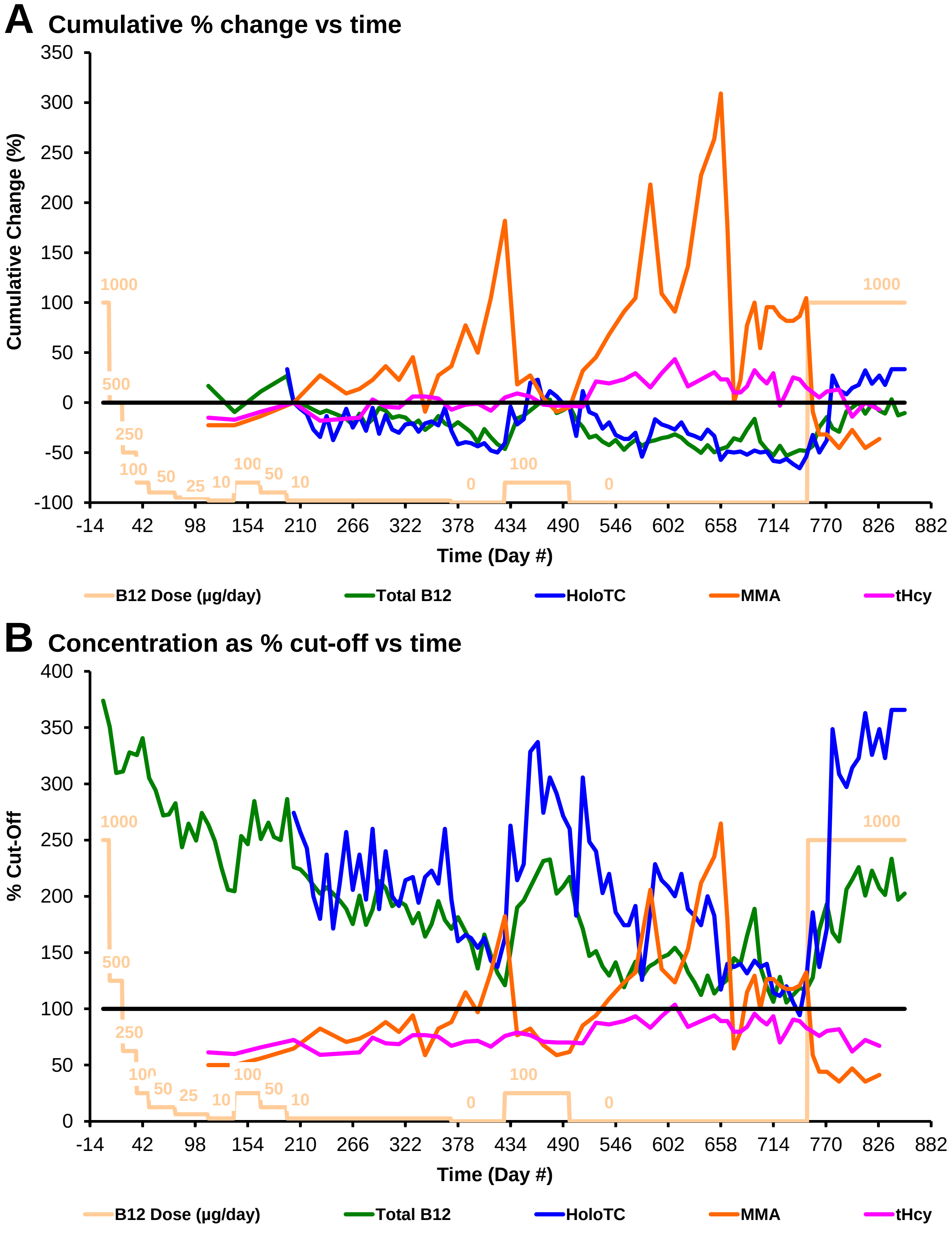
A
Cumulative % change vs time
Cumulative Change (%)
350
300
250
200
150
100
50
0
-50
-100
-14
42
98
154
210
266
322
378
434
490
546
602
658
714
770
826
882
Time (Day #)
1000
500
250
100
50
25
10
100
50
10
0
100
0
1000
B12 Dose (µg/day)
Total B12
HoloTC
MMA
tHcy
B
Concentration as % cut-off vs time
% Cut-Off
400
350
300
250
200
150
100
50
0
-14
42
98
154
210
266
322
378
434
490
546
602
658
714
770
826
882
Time (Day #)
1000
500
250
100
50
25
10
100
50
10
0
100
0
1000
B12 Dose (µg/day)
Total B12
HoloTC
MMA
tHcy

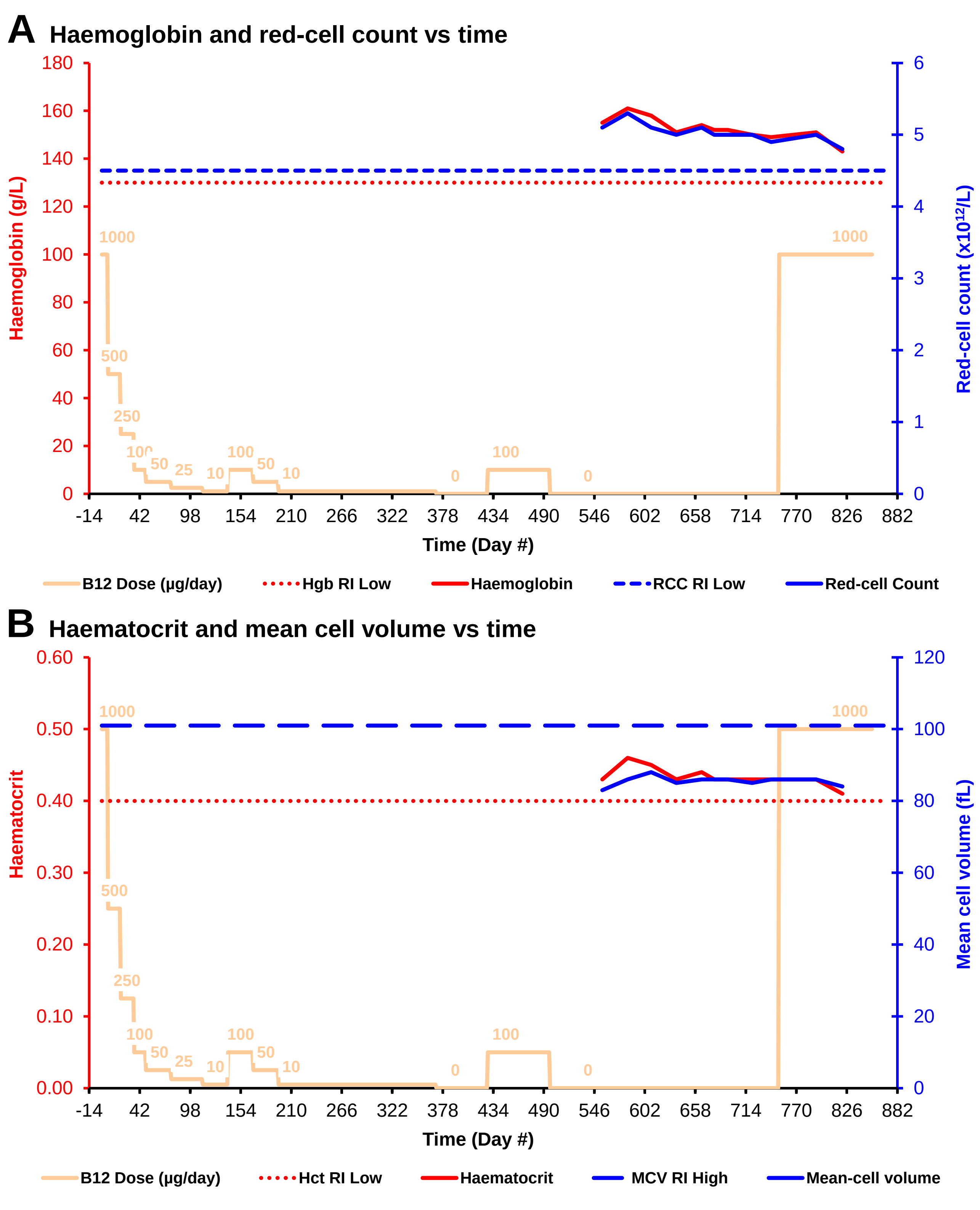
A
Haemoglobin and red-cell count vs time
Haemoglobin (g/L)
Red-cell count (x10^12/L)
Time (Day #)
1000
500
250
100
50
25
10
100
50
10
0
100
0
1000
B12 Dose (µg/day)
Hgb RI Low
Haemoglobin
RCC RI Low
Red-cell Count
B
Haematocrit and mean cell volume vs time
Haematocrit
Mean cell volume (fL)
Time (Day #)
B12 Dose (µg/day)
Hct RI Low
Haematocrit
MCV RI High
Mean-cell volume
