# Supplementary figures and images for: Experimental vitamin B12 deficiency in a human subject: a longitudinal investigation of the performance of the holotranscobalamin (HoloTC, Active-B12) immunoassay
Source: Springerplus. 2016 Feb 25;5:184. doi: 10.1186/s40064-016-1740-5 (PMC4767712; doi:10.1186/s40064-016-1740-5)

## Slide 1
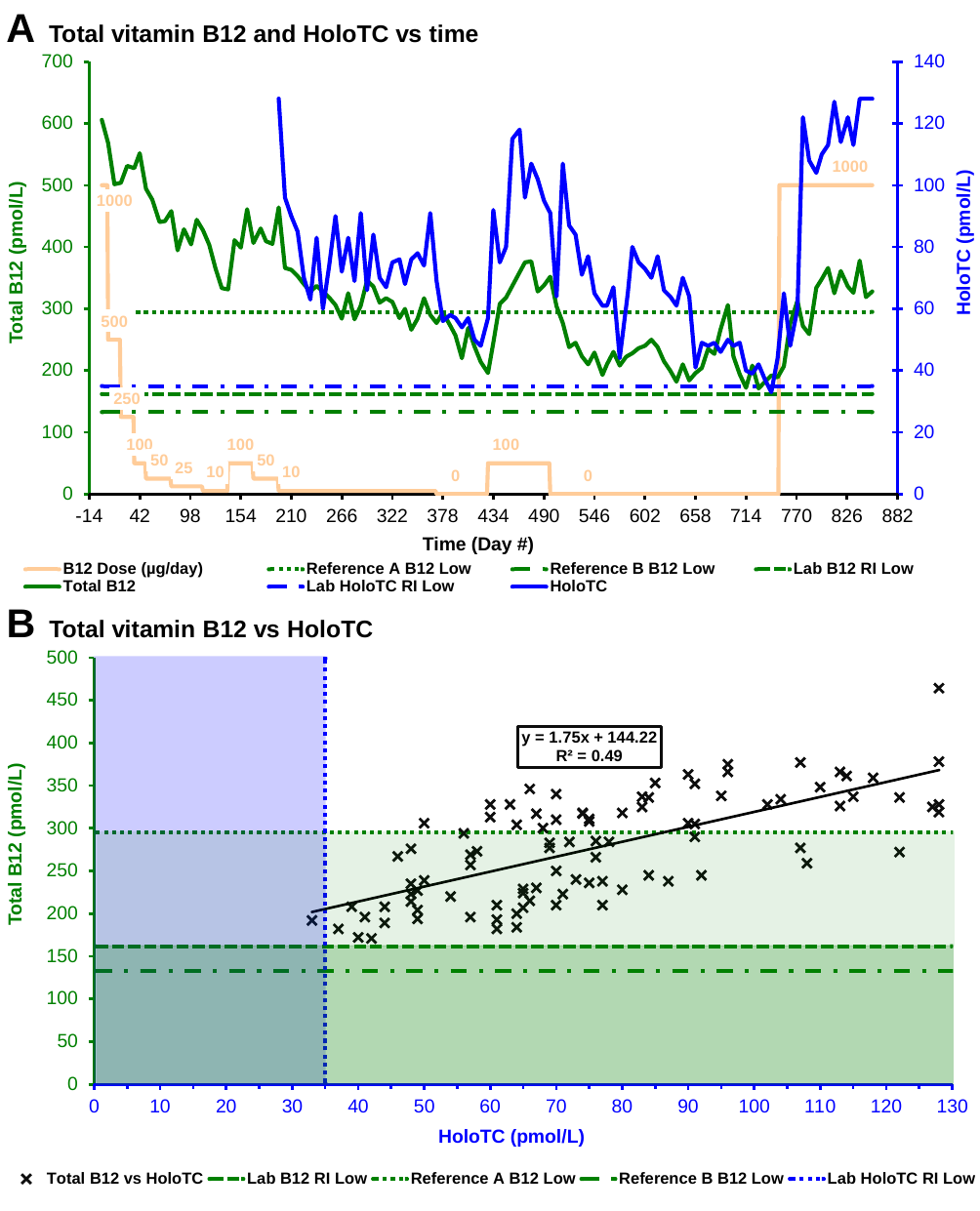

## Slide 2
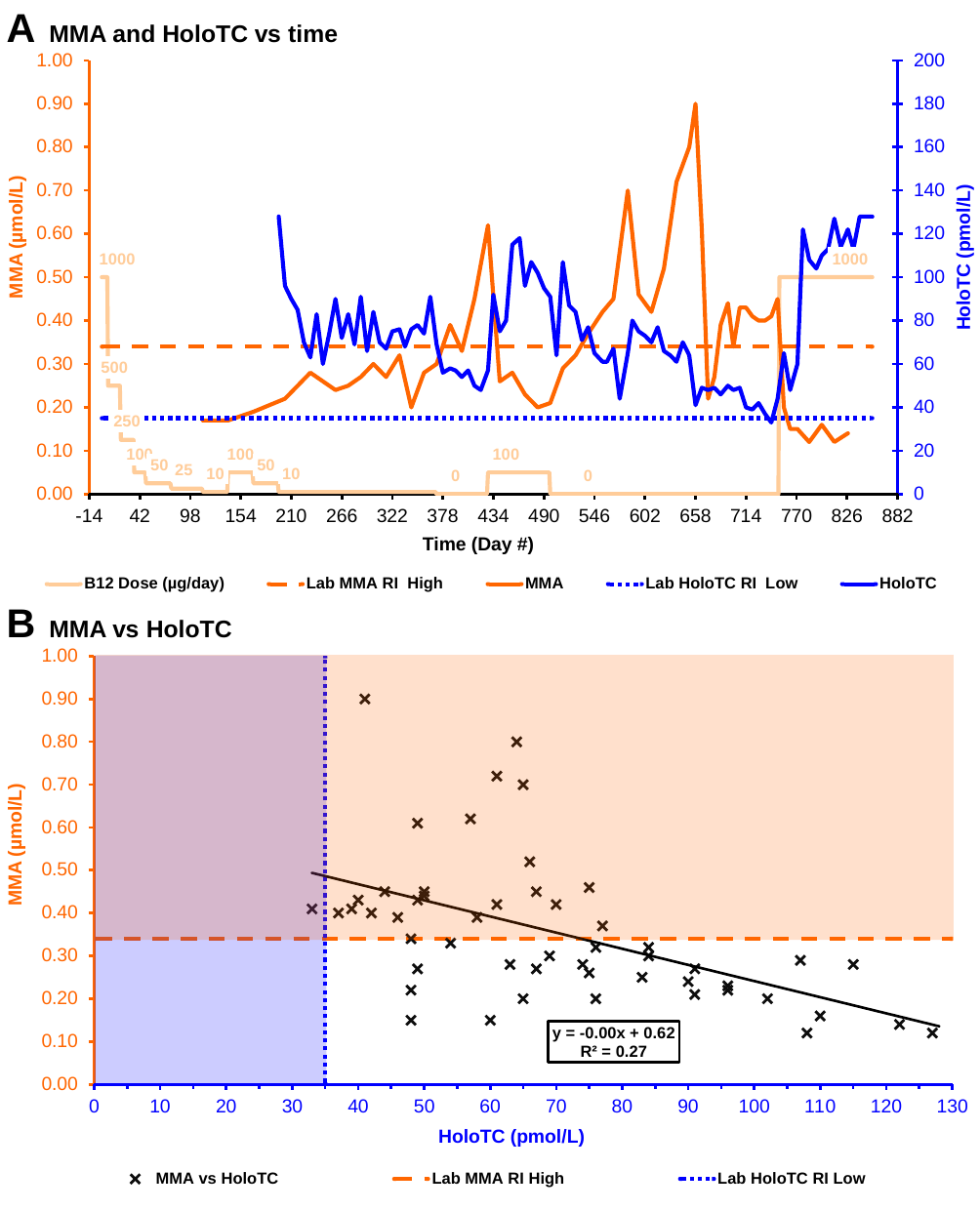

## Slide 3
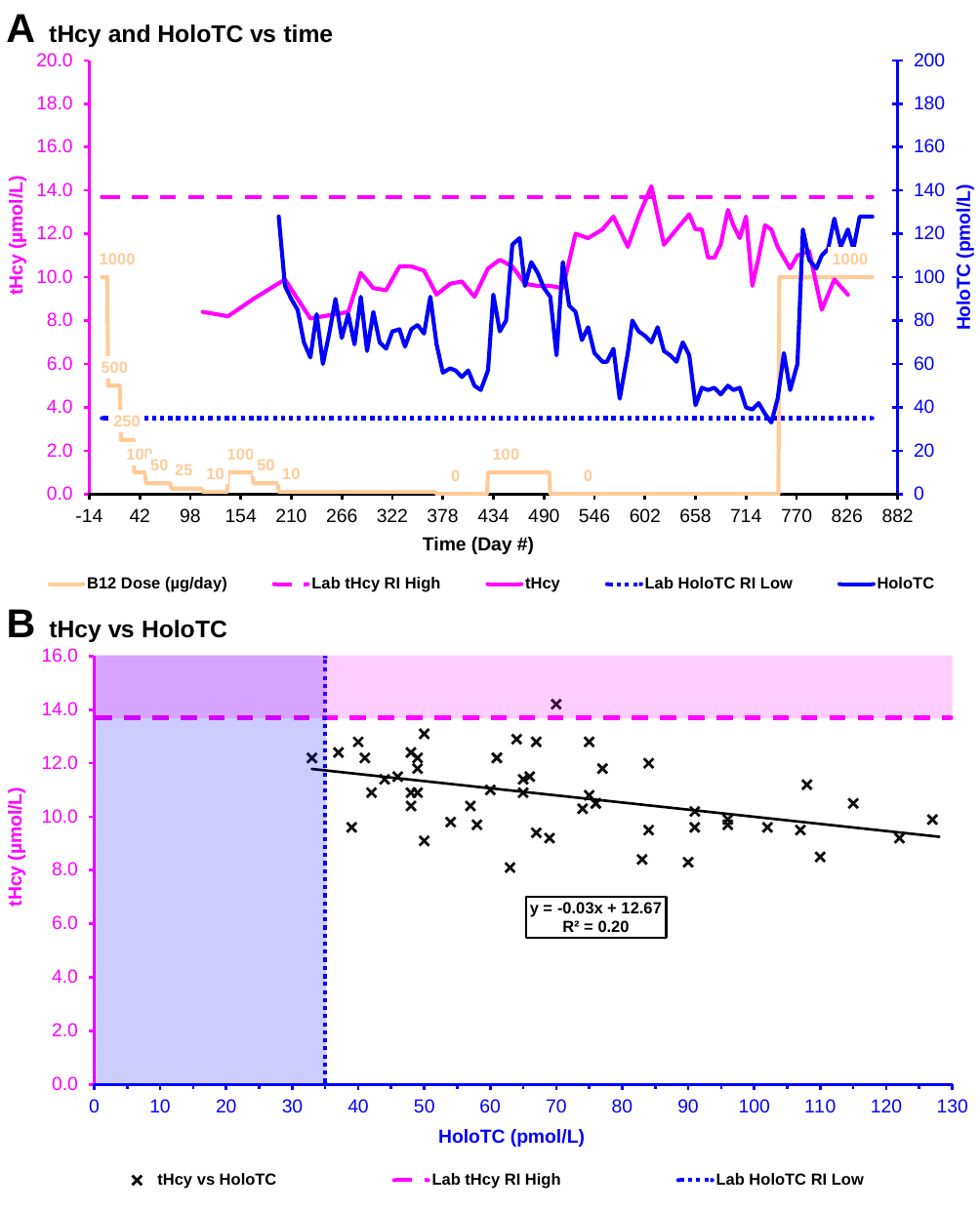

## Slide 4
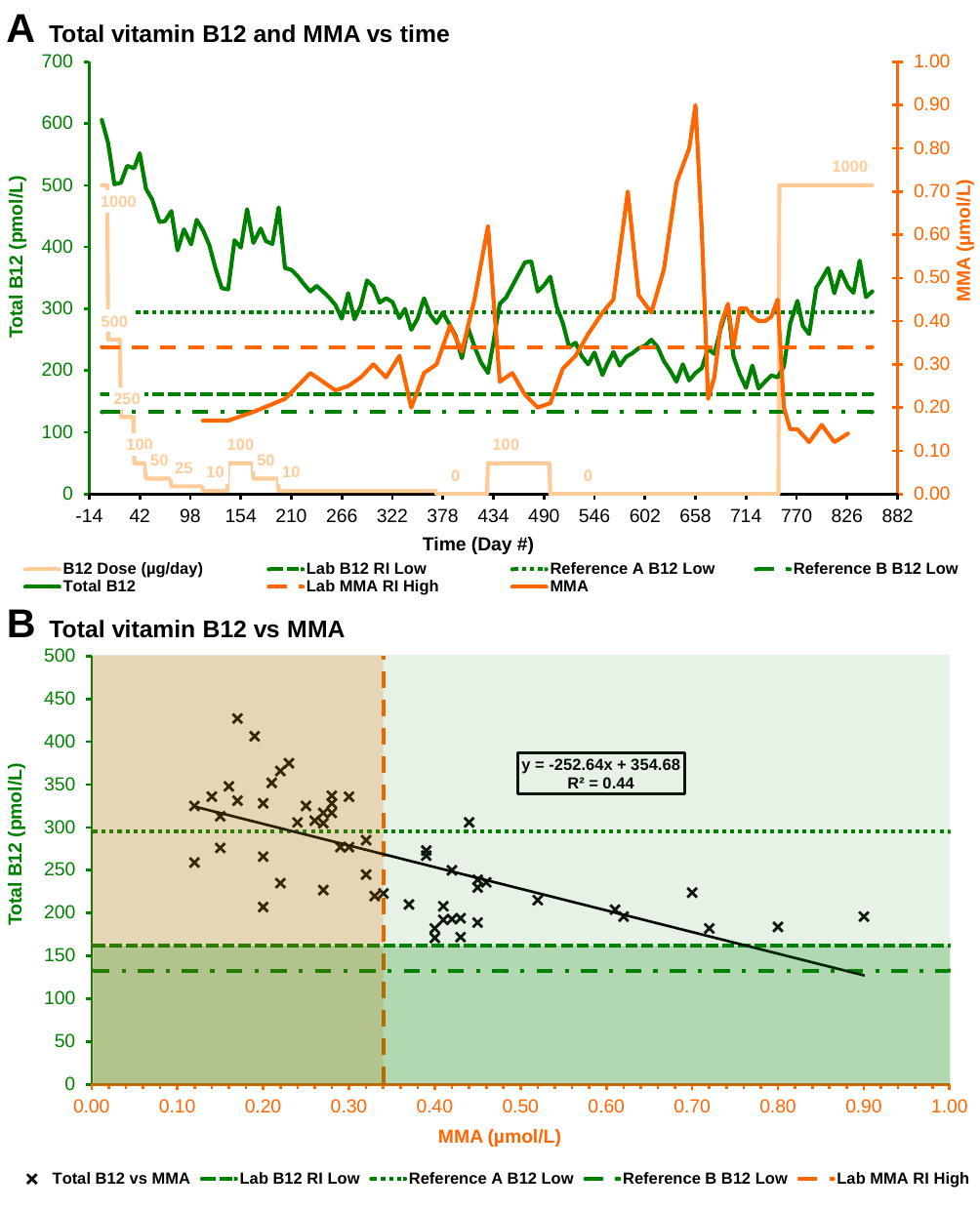

## Slide 5
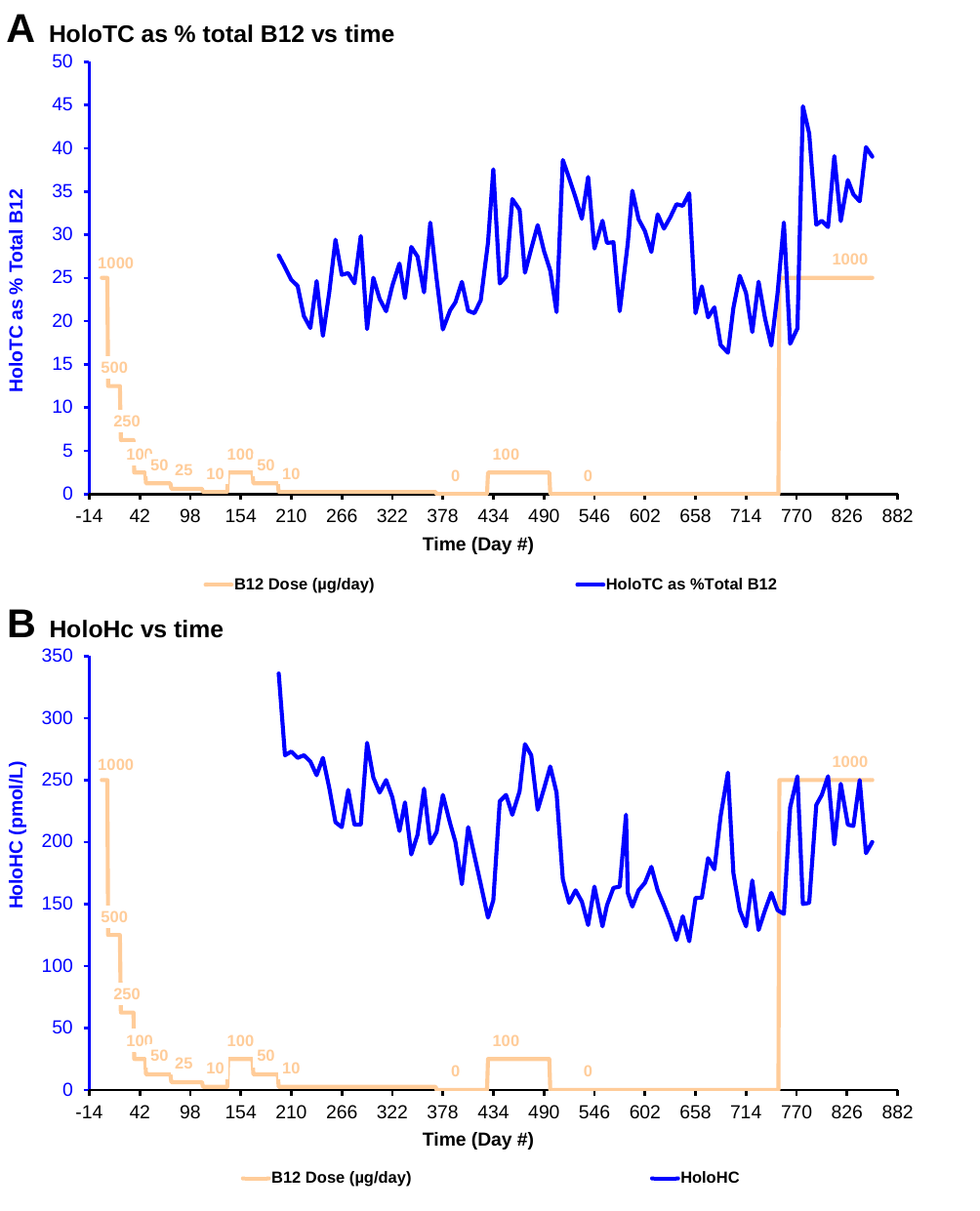

## Slide 6
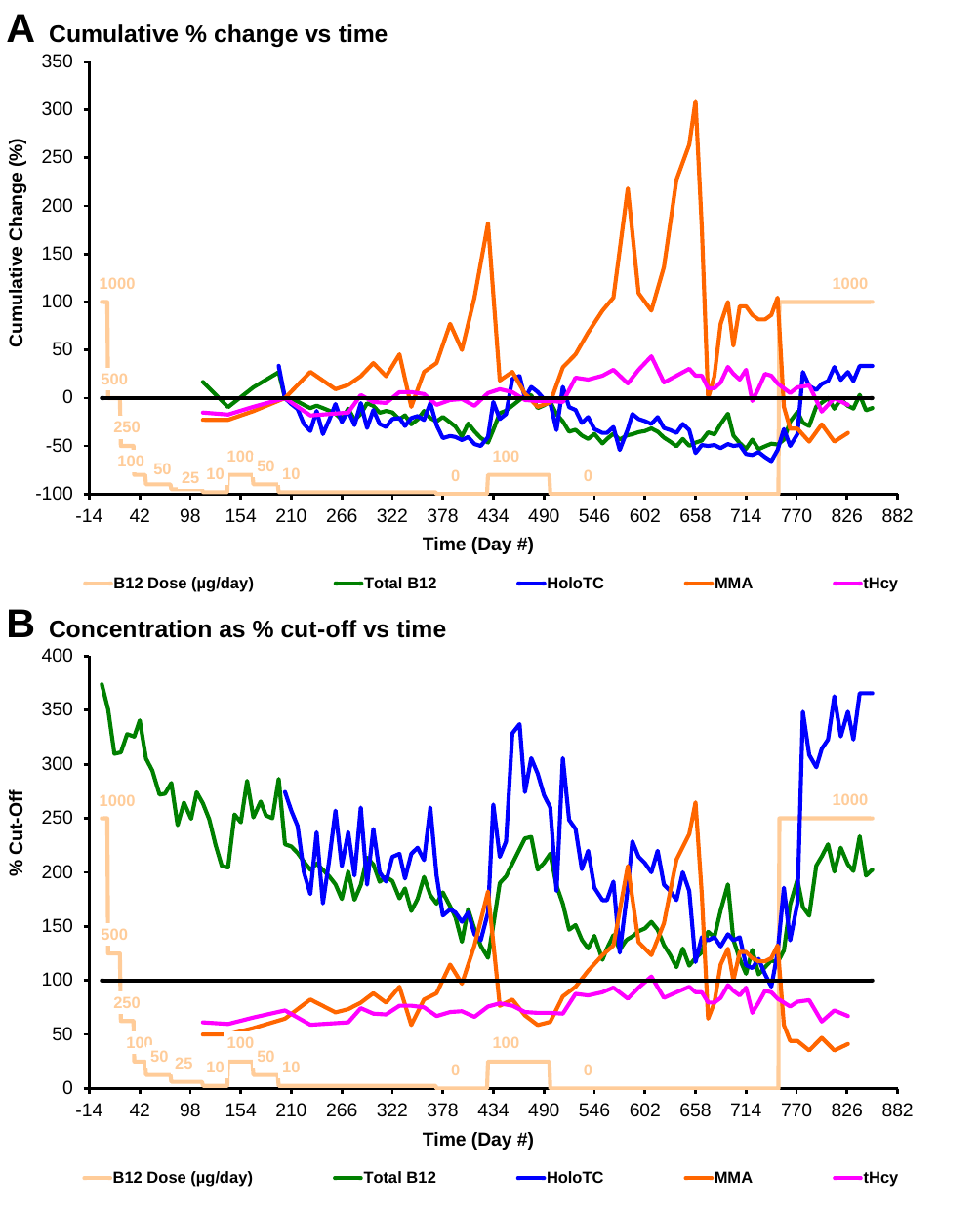

## Slide 7
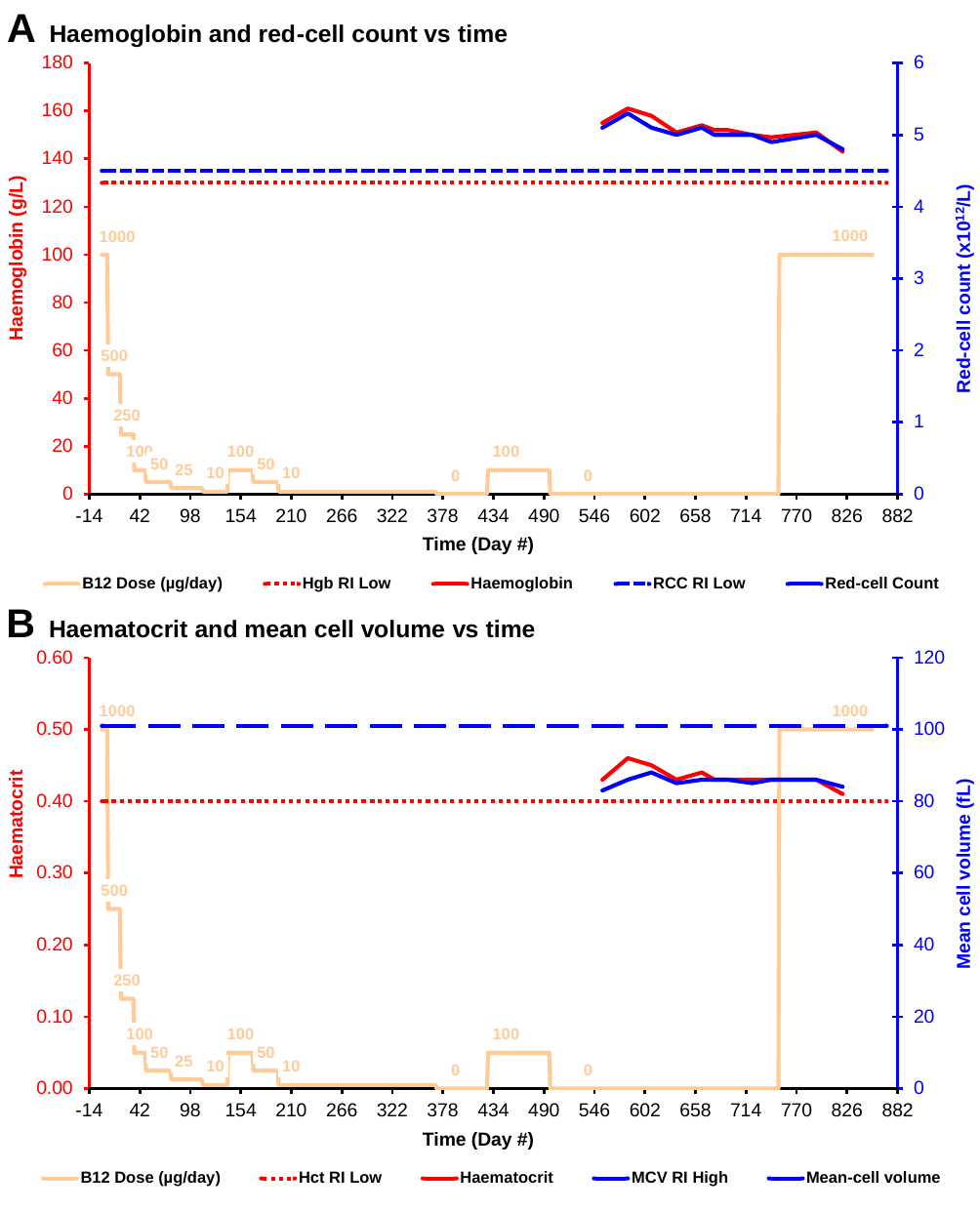

Supplement: Supplementary file 3 — 10.1186/s40064-016-1740-5 Figures 1 to 7, High-resolution slides. [file 40064_2016_1740_MOESM3_ESM.pptx]
